# Senolytic‐Resistant Senescent Cells Have a Distinct SASP Profile and Functional Impact: The Path to Developing Senosensitizers

**DOI:** 10.1111/acel.70358

**Published:** 2025-12-29

**Authors:** Utkarsh Tripathi, Masayoshi Suda, Vagisha Kulshreshtha, Bryan T. Piatkowski, Allyson K. Palmer, Nino Giorgadze, Christina Inman, Nathan Gasek, Ming Xu, Kurt O. Johnson, Tamar Pirtskhalava, Selim Chaib, Larissa P. G. Langhi Prata, Yi Zhu, Renuka Kandhaya‐Pillai, Stefan G. Tullius, Saranya P. Wyles, Rambabu Majji, Hari Krishna Yalamanchili, David B. Allison, Tamar Tchkonia, James L. Kirkland

**Affiliations:** ^1^ Department of Physiology and Biomedical Engineering Mayo Clinic Rochester Minnesota USA; ^2^ Department of Molecular Pharmacology and Experimental Therapeutics Mayo Clinic Rochester Minnesota USA; ^3^ Center for Advanced Gerotherapeutics, Division of Endocrinology and Metabolism Cedars‐Sinai Medical Center Los Angeles California USA; ^4^ Department of Cardiovascular Biology and Medicine Juntendo University Graduate School of Medicine Bunkyo‐ku Tokyo Japan; ^5^ Mayo Clinic Graduate School of Biomedical Sciences, Mayo Clinic Rochester Minnesota USA; ^6^ Department of Quantitative Health Sciences Mayo Clinic Rochester Minnesota USA; ^7^ Division of Hospital Internal Medicine, Department of Medicine Mayo Clinic Rochester Minnesota USA; ^8^ Robert and Arlene Kogod Center on Aging, Mayo Clinic Rochester Minnesota USA; ^9^ University of Connecticut Farmington Connecticut USA; ^10^ Department of Cellular and Integrative Physiology Sam and Ann Barshop Institute for Longevity and Aging Studies, University of Texas Health Science Center at San Antonio San Antonio Texas USA; ^11^ Department of Laboratory Medicine and Pathology University of Washington Seattle Washington USA; ^12^ Division of Transplant Surgery, Department of Surgery Brigham and Women's Hospital, Harvard Medical School Boston Massachusetts USA; ^13^ Department of Dermatology Mayo Clinic Rochester Minnesota USA; ^14^ Jan and Dan Duncan Neurological Research Institute, Texas Children's Hospital Houston Texas USA; ^15^ Department of Pediatrics USDA/ARS Children's Nutrition Research Center, Baylor College of Medicine Houston Texas USA; ^16^ Department of Epidemiology and Biostatistics Indiana University‐Bloomington Bloomington Indiana USA

**Keywords:** cellular senescence, senescent cell subtypes, senolytics, senosensitizers

## Abstract

The senescent cell (SC) fate is linked to aging, multiple disorders and diseases, and physical dysfunction. Senolytics, agents that selectively eliminate 30%–70% of SCs, act by transiently disabling the senescent cell antiapoptotic pathways (SCAPs), which defend those SCs that are proapoptotic and pro‐inflammatory from their own senescence‐associated secretory phenotype (SASP). Consistent with this, a JAK/STAT inhibitor, Ruxolitinib, which attenuates the pro‐inflammatory SASP of senescent human preadipocytes, caused them to become “senolytic‐resistant”. Administering senolytics to obese mice selectively decreased the abundance of the subset of SCs that is pro‐inflammatory. In cell cultures, the 30%–70% of human senescent preadipocytes or human umbilical vein endothelial cells (HUVECs) that are senolytic‐resistant (to Dasatinib or Quercetin, respectively) had increased p16^INK4a^, p21^CIP1^, senescence‐associated β‐galactosidase (SAβgal), γH2AX, and proliferative arrest similarly to the total SC population (comprising senolytic‐sensitive plus‐resistant SCs). However, the SASP of senolytic‐resistant SCs entailed less pro‐inflammatory/apoptotic factor production, induced less inflammation in non‐senescent cells, and was equivalent or richer in growth/fibrotic factors. Senolytic‐resistant SCs released less mitochondrial DNA (mtDNA) and more highly expressed the anti‐inflammatory immune evasion signal, glycoprotein non‐melanoma‐B (GPNMB). Transplanting senolytic‐resistant SCs intraperitoneally into younger mice caused less physical dysfunction than transplanting the total SC population. Because Ruxolitinib attenuates SC release of proapoptotic SASP factors, while pathogen‐associated molecular pattern factors (PAMPs) can amplify the release of these factors rapidly (acting as “senosensitizers”), senolytic‐resistant and senolytic‐sensitive SCs appear to be interconvertible.

## Graphical abstract

The SC subtype with a pro‐inflammatory, proapoptotic, tissue‐damaging SASP is susceptible to senolytics. The SC subset not killed by senolytics has a pro‐growth/repair SASP. “Senosensitizers” that induce senolytic‐resistant SCs to be converted into SCs that are susceptible to senolytics could enable removal of relatively silent SCs.

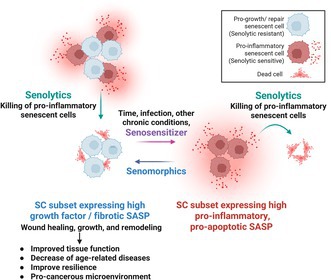

## 1 Introduction

The senescent cell (SC) fate is linked to aging, multiple disorders and diseases, and physical dysfunction (Wyles et al. 2022; Palmer et al. 2022; Wyld et al. 2020; Muñoz‐Espín and Serrano 2014; He and Sharpless 2017; Tchkonia et al. 2021; Wissler Gerdes, Zhu, Weigand, et al. 2020; Khosla et al. 2020; Suda, Paul, et al. 2023). Senescence can occur in most cell types across the vertebrates and can be induced by replicative stress, DNA damage, cytotoxic drugs, radiation, inflammation, metabolic dysfunction, pathogen exposure, and other insults (Wyles et al. 2022; Palmer et al. 2022; Muñoz‐Espín and Serrano 2014; He and Sharpless 2017; Khosla et al. 2020; Suda et al. 2024). Senescence entails resistance to apoptosis and SCs are generally removed by innate and adaptive immune system components (Wang et al. 2022; Prata et al. 2018; Katsuumi et al. 2024). Senescence‐associated cell cycle arrest can serve to curtail the proliferation of dysfunctional, damaged, or precancerous cells (Campisi 2005). In preclinical cell and tissue culture and animal models, it appears that while senescence contributes to a range of pathological processes, this cell fate has essential roles, such as for cancer prevention, embryonic development and parturition, and wound healing (Wyld et al. 2020; Wang et al. 2022; Demaria 2014; Born et al. 2023; Kirkland 2023; Cubro et al. 2021). However, those persisting SCs that are not removed by the immune system can develop a tissue‐destructive SASP, spread senescence locally and systemically, develop and harbor potentially cancerous mutations, and can lead to deleterious sequelae, including tumor development, chronic inflammation, immune deficits, and progenitor cell dysfunction (Suda et al. 2024; Kirkland 2023; Gorbunova et al. 2021; Kale et al. 2020; Stout et al. 2014; Tchkonia et al. 2010; Chaib et al. 2024). Consistent with this, transplanting small numbers of SCs can result in frailty, osteoarthritis, and accelerated death from most of the diseases that occur later in life in naturally aged mice (Xu et al. 2018, 2017).

SCs are not homogeneous: they exhibit significant heterogeneity in their characteristics and behavior depending on the type of cell that became senescent, the inducer of senescence, time since senescence was induced, host *milieu*, and disease states, and arguably, SCs may be of pro‐inflammatory “deleterious” or reparative “helper” subtypes (Suda, Paul, et al. 2023; Tripathi, Misra, et al. 2021; Kirkland and Tchkonia 2020; De Cecco et al. 2019; Tripathi, Nchioua, et al. 2021; Suda et al. 2025). There are challenges in examining the heterogeneity of SCs across human tissues due to the low abundance of SCs and controversy about sensitive and specific markers of senescence (Gasek et al. 2021).

Senolytics are agents that were originally developed using a hypothesis‐driven, mechanism‐based approach to target and eliminate senescent cells selectively: the first senolytics were designed to transiently disable the SCAPs that protect the subset of SCs that are pro‐apoptotic from being targeted and removed by their own SASP (Zhu et al. 2015, 2017; Wissler Gerdes, Zhu, Tchkonia, and Kirkland 2020). We previously demonstrated that the first senolytics reported, Dasatinib and Quercetin, eliminate 30%–70% of senescent cells through apoptosis (terminal deoxynucleotidyl transferase dUTP nick end labeling [TUNEL] assay; figures 2E,F in Zhu et al. (2015)) and the percent of cleaved caspase 3^+^ cells (figure 4E in Xu et al. (2018)). Although Dasatinib and Quercetin remove 30%–70% of SCs, senolytics still delay, prevent, alleviate, or treat multiple disorders and diseases across the lifespan in preclinical models (Xu et al. 2018, 2021; Zhu et al. 2015; Zhang et al. 2019; Roos et al. 2016; Farr et al. 2017; Schafer et al. 2017; Moncsek et al. 2018; Saccon et al. 2021; Ogrodnik et al. 2019; Suda, Katsuumi, et al. 2023). However, there are few data about the nature and role of the “senolytic‐resistant” SCs that remain after senolytic exposure versus “senolytic‐sensitive” SCs. Here, we explore mechanisms through which those SCs that are sensitive versus resistant to senolytics can be interconverted and their impact in vivo by transplanting these two senescent cell subtypes into younger mice.

## 2 Materials and Methods

### 2.1 Cell Culture

**FIGURE 1:**
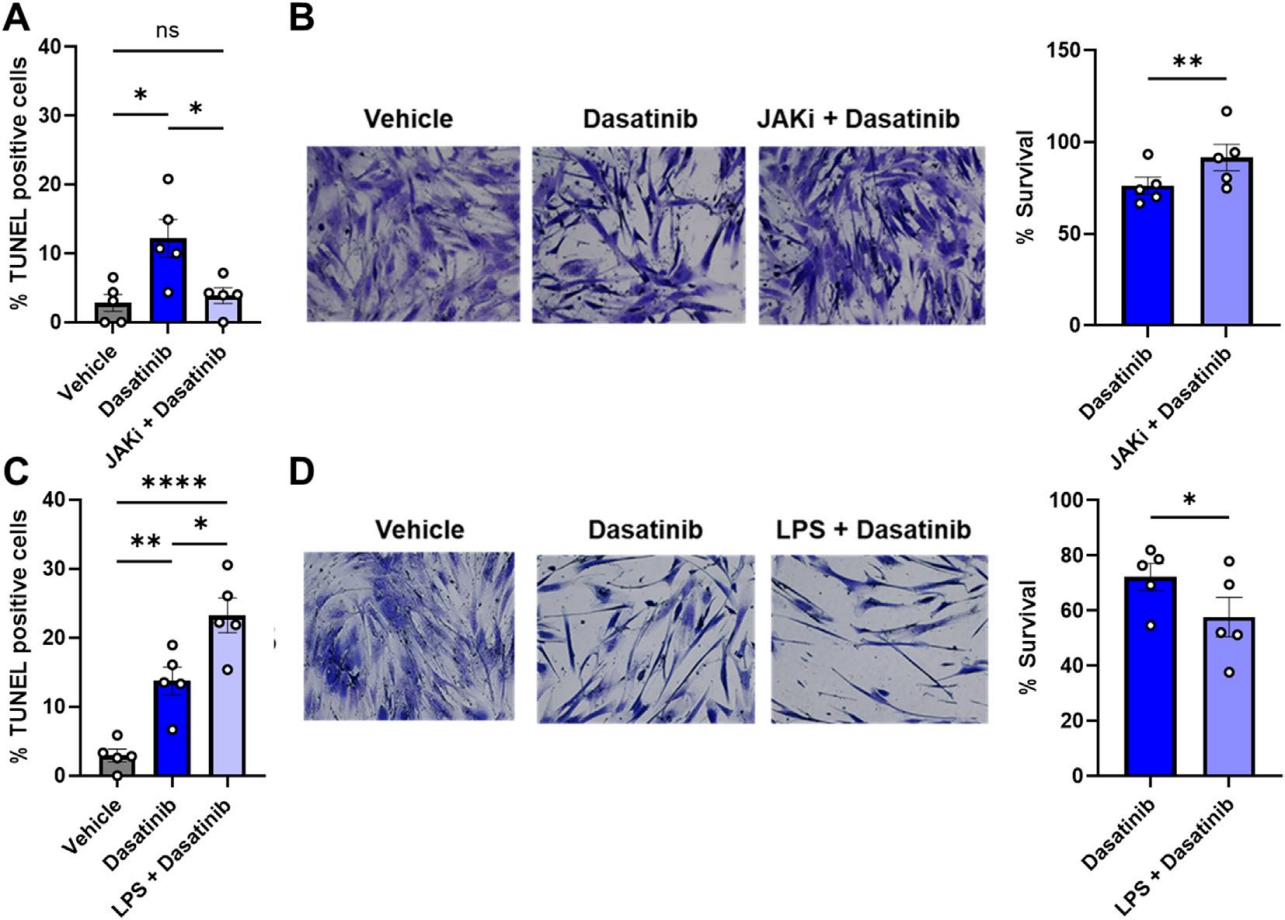
The SASP impacts extent of SC clearance by senolytics. (A) TUNEL‐positive nuclei as a percent of total cells and (B) representative images of surviving (crystal violet^+^) senescent preadipocytes and quantification relative to vehicle‐treated cells after exposure to vehicle or Ruxolitinib (1 μM), which attenuates the pro‐inflammatory SASP, for 3 days followed by Dasatinib 800 nM for 24 h. (C) TUNEL‐positive nuclei as a percent of total cells and (D) survival of SCs pre‐treated with 10 μg/mL LPS for 3 days followed by treatment with Dasatinib 800 nM for 24 h. Quantification of images (*N* = 5) is shown on the right. Data are shown as means +/− SEM; 1‐way ANOVA; and post hoc comparisons with by Tukey's HSD multiple comparison (A, C), and are expressed as a function of vehicle‐treated cells; means +/− SEM; paired, 2‐tailed Student's *t*‐tests.

Preadipocytes (also known as adipose‐derived stromal cells or adipose mesenchymal “stem” cells [MSCs]) were isolated from abdominal subcutaneous fat biopsies obtained from subjects donating kidneys for transplantation aged 38.4 +/− 11.4 years (mean +/− SD), median 37 (25–68), with a body mass index 28.3 +/− 3.9 (mean +/− SD), median 27.2 (23–40.46) or undergoing bariatric surgery aged 39.25 +/− 8.5 years (mean +/− SD), median 40.5 (28–48) with a body mass index 39.7 +/− 5.03 (mean +/− SD), median 37.29 (36.6–47.1), both males and females. We primarily used preadipocytes obtained from kidney transplant patients. Preadipocytes from both kidney transplant and bariatric surgery patients were used in Figures 1, 4, and 5. All subjects gave informed consent. The protocol was approved by the Mayo Clinic Institutional Review Board for Human Research. Using methods we previously reported (Tripathi, Nchioua, et al. 2021): (1) human preadipocytes were isolated and cultured; (2) all human preadipocyte lots that were not induced to become senescent retained proliferative capacity over multiple passages with low markers of senescence, and were therefore considered to be non‐senescent/control cells; and (3) human preadipocyte senescence was induced by 20 Gy X‐irradiation (X‐Rad320, PRECISION) and experiments were performed 30 days after radiation. At this time point, more than 70% of cells were positive for SA‐β‐gal staining, exhibited minimal proliferation, and had markedly elevated expression of SASP factors (Tripathi, Nchioua, et al. 2021). To minimize inter‐donor variability, we employed a paired experimental design in Figures 1B,D, 4E, and 5B, Figure S4C,D. Preadipocytes from each donor were divided into three groups: one served as a non‐irradiated control (non‐senescent) and the others were subjected to irradiation to induce senescence followed by vehicle or senolytic treatment. In HUVECs purchased from Lonza (CC‐2519, San Diego, California), senescence was induced by 10 Gy X‐irradiation and experiments were conducted 10–14 days after irradiation. The MDA‐MB‐231 human breast cancer cell line was obtained from ATCC. Cells were treated with 1 μ/mL Cisplatin for 24 h and maintained in culture for 1 week. The cells were then treated with Dasatinib (200 nM) or vehicle for 24 h followed 24 h later by a TLR3 agonist, polyinosine‐polycytidylic acid (10 μg/mL), or vehicle daily for 3 days. After treatment with the TLR3 agonist or vehicle, cells were again exposed to Dasatinib or vehicle for 24 h.

### 2.2 Reagents

Dasatinib (SML2589) and Quercetin (Q4951) were purchased from Sigma (Sigma, St. Louis, MO, USA). The JAK inhibitor Ruxolitinib (tlrl‐rux) was purchased from InvivoGen (Sigma, St. Louis, MO, USA). Lipopolysaccharide (LPS; tlrl‐3pelps) was purchased from InvivoGen (San Diego, CA, USA).

### 2.3 Rt‐PCR and RNA Sequencing

For most studies, cells were washed with PBS and RNA was isolated using Trizol and chloroform (Xu et al. 2015). Concentration and purity of samples were assayed using a Nanodrop spectrophotometer. Each cDNA sample was generated by reverse transcription using 1–2000 ng RNA following the manufacturer's recommended protocol (High‐capacity cDNA Reverse Transcription Kit; Cat #4368813, Thermo Fisher Scientific, Waltham, MA, USA). A reverse transcription program was used (10 min at 25°C, 120 min at 37°C, 5 min at 85°C, held at 4°C). TATA‐box binding protein (TBP) served as a control for gene expression analyses. A list of primers is provided in Table 2. For RNA sequencing, library preparation was performed using a TruSeq Stranded mRNA kit prior to sequencing on the Illumina NovaSeq 6000 platform.

Raw reads were trimmed with fastp (v0.20.1) (Bolger et al. 2014). Trimmed reads were then mapped to the reference genome GRCh38 with STAR (v2.7.9a) (Kim et al. 2015) and gene expression is estimated by generating a count matrix with STAR‐counts. The raw gene counts were adjusted for the sample pairing covariate using ComBat‐Seq within the R package, sva v3.50.0 (Zhang et al. 2020). Differential expression analysis was performed using the R package, edgeR v4.0.16 (Robinson et al. 2009). Raw gene counts were first pre‐filtered to keep only those genes with greater than one average count per million in either comparison group. The raw *p* values were adjusted using the Benjamini–Hochberg procedure (Benjamini and Hochberg 1995) to control the false discovery rate, and those genes with an adjusted *p* value ≤ 0.05 were considered to be significant. Differentially expressed genes were used for heatmap visualization and hierarchical clustering. Functional enrichment analyses were performed using clusterProfiler v4.14.0 (Wu et al. 2021).

### 2.4 Conditioned Media (CM)

CM were prepared by exposing cells to RMPI 1640 containing 1 mM sodium pyruvate, 2 mM glutamine, MEM (minimum essential medium) vitamins, MEM nonessential amino acids (all Gibco), and Streptomycin (Life Technologies, Carlsbad, CA, USA) (Xu et al. 2015). CM were collected 24–48 h after the cells had been exposed to the media.

### 2.5 TUNEL Assay

Cellular apoptosis was assessed using the In Situ Cell Death Detection Kit (Roche, 11684795910) according to the manufacturer's instructions. Samples were counterstained with DAPI (Life Technologies). Apoptotic and nonapoptotic cells were examined by fluorescence microscopy (Nikon ECLIPSE Ti, NIS Elements AR 5,20,02).

### 2.6 Cell Survival

Cell survival was assayed by crystal violet. Briefly, cells were washed with PBS twice and fixed with 4% PFA in PBS for 15 min and then washed again with PBS after incubation. Cells were then treated with 0.5% crystal violet in 25% methanol. After incubation for 10 min, crystal violet was removed and plates were washed with water until washings were clear of visible dye. Plates were dried and crystal violet was dissolved in 100% methanol. Absorbance at 570 nM was quantified.

### 2.7 SAβgal Assay

SAβgal activity was assayed as previously described (Baker et al. 2011). In brief, primary preadipocytes were washed with PBS and then fixed for 5 min in PBS containing 2% (vol/vol) formaldehyde (Sigma‐Aldrich, St. Louis, USA) and 0.25% glutaraldehyde (Sigma‐Aldrich, St. Louis, USA). Following fixation, cells or tissues were washed with PBS before being incubated in SAβgal activity solution (pH 6.0) at 37°C for 16–18 h. The enzymatic reaction was stopped by washing cells or tissues 3–5X with ice‐cold PBS. Bright field microscopy (Nikon Eclipse Ti) was used for imaging.

### 2.8 mtDNA Assay

Total DNA was extracted from media using the QIAmp‐Blood & Tissue kit (Qiagen, Germantown, MD, USA). mtDNA copy number was measured by quantitative rtPCR using primers for mitochondrially encoded NADH dehydrogenase subunit 2 (MT‐ND2) and cytochrome c oxidase subunit III (MT‐COX3) of the electron transport chain.

### 2.9 Immunostaining

Cells were fixed with 4% (vol/vol) paraformaldehyde for 10 min and permeabilized using PBS containing 0.25% Triton X‐100 for 10 min. After being washed with PBS, cells were blocked with PBS and Tween‐20 (PBST) containing 1% BSA for 30 min. Cells were incubated with primary antibodies in 1% BSA in PBST (blocking buffer) overnight at 4°C, washed with PBS and then incubated with secondary fluorescent antibodies (Life Technologies, Carlsbad, CA, USA) in the blocking buffer for 1 h in the dark. DAPI (Life Technologies, Carlsbad, CA, USA) was used to stain nuclei for cell counting. Fluorescence microscopy (Nikon Eclipse Ti) was used for imaging. For EdU incorporation assays, cells were incubated in 10 μM EdU for 24 h in growth medium. All subsequent EdU detection steps were carried out using Click‐iT EdU Alexa Fluor Imaging Kits (C10337, Thermo Fisher Scientific, Waltham, MA USA) according to the manufacturer's instructions. The following antibodies were used: p16 (1:200 dilution, Cat# 705‐7493, Cintech Histology); p21 (1:100 dilution, Cat# 109199, Abcam, Cambridge, MA); and Phospho‐Histone H2A.X (Ser139) (20E3) (Rabbit; 1:200 dilution, Cat #9718, Cell Signaling, Danvers, MA).

### 2.10 Mice

Two‐month‐old *SCID‐beige* mice (Charles River Laboratories, Wilmington, MA, USA) were used for the SC transplantation studies. Mice were maintained in a pathogen‐free facility at 23°C–24°C under a 12‐h light, 12 h dark regimen with free access to water and a chow diet (standard mouse diet with 20% protein, 5% fat [13.2% fat by calories], and 6% fiber; Lab Diet 5053, St. Louis, MO). For the obese mouse studies, animals were maintained on a 60% (by calories) fat diet (D12492, irradiated; Research Diets, New Brunswick, NJ). All mouse studies were approved by the Mayo Clinic Institutional Animal Care and Use Committee.

### 2.11 Cell Transplantation

Human preadipocyte senescence was induced by 20 Gy x‐irradiation. SCs or control nonirradiated cells were collected by trypsinization. Cell pellets were washed with PBS once and resuspended in PBS for transplantation. Recipient *SCID‐beige* mice were anesthetized using isoflurane and cells were injected intraperitoneally in 150–200 μL PBS through a 22 G needle (Xu et al. 2018).

### 2.12 Physical Function Assays

Forelimb grip strength was determined in the transplanted mice using a grip strength meter (Columbus Instruments, Columbus, Ohio) (Xu et al. 2018). Results were averaged from 3 to 5 trials. For the wire hanging test (Xu et al. 2018), mice were placed on a 2 mm thick metal wire, which was placed 35 cm above a padded surface. Mice were allowed to grab the wire only with their forelimbs. Hanging time was normalized to body weight as hanging duration in seconds × body weight (grams). Results were averaged from 3 trials for each mouse. In this experiment, mice were housed in cages. This may introduce clustering effects due to the dependency of the mice in the same group. The authors acknowledge the need to properly account for clustering due to the risk of underestimating standard errors and inflating the Type I error rates. However, we do not expect a significant impact of the clustering effects in this study because animals from all three groups (treated with non‐senescent cells, senescent cells, and senolytic‐resistant senescent cells) were together in the same cages. Thus, any within‐cluster effects on the variability of the outcome would impact all mice rather than a specific mouse group. Additionally, findings rely more on treatment group responses, not individual mouse responses.

### 2.13 Multiplex ELISA

CM were filtered and cytokine and chemokine protein levels in CM were assayed using Luminex xMAP technology. Multiplexing analysis was performed using a Luminex 100 system (Luminex, Austin, TX, USA) by Eve Technologies Corp. (Calgary, Alberta, Canada). Data are represented as pg/ml for each SASP factor as a function of cellular density.

### 2.14 Mass Cytometry by Time of Flight (CyTOF)

An antibody panel was designed based on surface markers, transcription factors, and cytokines. Each antibody was tagged with a rare metal isotope (Table 1) and function verified by mass cytometry according to the manufacturer's manual (Multi Metal labeling kits; Fluidigm South San Francisco, CA, USA). A CyTOF‐2 mass cytometer (Fluidigm, South San Francisco, CA, USA) was used for data acquisition. Acquired data were normalized based on normalization beads (Ce140, Eu151, Eu153, Ho165, and Lu175 as in (Palmer et al. 2019)). Preadipocytes were isolated from mouse epididymal white adipose tissue. Collected cells were incubated with metal‐conjugated antibodies for testing intracellular proteins including transcription factors and cytokines. Fixation and permeabilization were conducted according to the manufacturer's instructions (Foxp3/Transcription Factor Staining Buffer Set, eBioscience, San Diego, CA, USA). CyTOF data were analyzed by Cytobank (Santa Clara, CA, USA). Cell populations were clustered and visualized using viSNE, an implementation of the t‐distributed stochastic neighbor embedding (t‐SNE) algorithm based on the Barnes‐Hut approximation, available within the Cytobank platform (Van Der Maaten 2014). Total preadipocytes were considered to be CD45^−^, CD31^−^, and Sca1^+^, macrophages F4/80^+^ and CD11b^+^, endothelial cells CD31^+^ and CD146^+^, and T lymphocytes CD4^+^ or CD8^+^ as in (Palmer et al. 2019).

**TABLE 1:** Antibodies used for CyTOF.

Label	Protein	Clone	Company
143 Nd	pS139 H2AX	N1‐431	BD Bioscience
144 Nd	p21	F‐5	Santa Cruz
145 Nd	CENP‐B	ab25734	Abcam
150 Nd	pS63 c‐Jun	9261	Cell Signalling
152 Sm	CD3e	145‐2C11	Fluidigm
153 Eu	CD8a	53‐6.7	Fluidigm
154 Sm	CD11b	M1/70	Fluidigm
155 Gd	IL‐6	MP5‐20F3	Biolegend
156 Gd	Activin A	69403	R&D Systems
158 Gd	PY705Stat3	4/P‐Stat3	Fluidigm
166 Er	CXCL1	MAB453	R & D Systems
170 Er	Flag	L5	Biolegend
171 Yb	IL10	JES5‐16E3	Biolegend
175 Lu	pS536 NF‐κB	93H1	Cell Signalling
176 Yb	TNFα	MP6‐XT22	Biolegend

**TABLE 2:** Primers used for rt‐PCR analyses.

*TBP*	Hs00427620_m1
*p16*	Hs00923894_m1
*p21*	Hs00355782_m1
*IL‐1α*	Hs00174092_m1
*CXCL1*	Hs00982282_m1
*CXCL5*	Hs00171085_m1
*IL‐1β*	Hs01555410_m1
*IL‐8*	Hs00174103_m1
*IL‐6*	Hs00174131_m1
*CCL2*	Hs00234140_m1
*CCL5*	Hs00982282_m1
*GPNMB*	Hs01095669_m1
*ND2*	Hs02596874_g1
*COX3*	F: 5′ CCACCAATCACATGCCTATCAT 3′
	R: 5′ GCTGAGAGGGCCCCTGTT 3′
	P: 5′ FAM_TAGTAAAACCCAGCCCATG_MGB 3′
*KRAS*	F: 5′ GGGAGTATGTCAGGGTCCATGA 3′
	R: 5′ CGAAACTCTGAAATACACTTCCAATC3′
	P: 5′ FAM _TTCACTCTCTGTGCATTT_ MGB 3′

### 2.15 Statistical Analyses

GraphPad Prism version 10.0.3 was used for statistical analyses. Results are presented as mean ± SD or SEM as stated in the figure legends. *p* < 0.05 was considered to be statistically significant and *p* < 0.05, *p* < 0.01, *p* < 0.005, and *p* < 0.001 are indicated as *, **, ***, and ****, respectively. Statistical analyses were performed using two‐tailed tests. Statistical tests include Student's *t*‐tests or two‐tailed Mann–Whitney tests comparing two groups, one‐way ANOVA and post hoc multiple comparison test with Tukey's Honestly Significant Difference (HSD) comparing more than two groups of pairs as detailed in the figure legends. Due to the limited sample size and the dependence of the methods used on the distributional assumptions of normality, a sensitivity analysis was then conducted using nonparametric tests based on taking the ranks of the variables then analyzing the ranks. Statistical significance of the results was diminished upon nonparametric testing as opposed to using standard *t*‐tests, supporting the desirability of conducting future confirmatory studies with larger sample sizes.

## 3 Results

### 3.1 The SASP Is Linked to Extent of SC Clearance by Senolytics

The JAK/STAT inhibitor (JAKi), Ruxolitinib, attenuates the SASP of senescent human preadipocytes (Xu et al. 2015). Reducing the SASP by Ruxolitinib or si‐RNA mediated knockdown of JAK1 attenuated the ability of Dasatinib to clear senescent human preadipocytes (Figure 1A,B, Figure S1A,B). Pathogen‐associated molecular pattern (PAMP) factors, such as lipopolysaccharide (LPS), which is in the outer membrane of gram‐negative bacteria, have been shown to exacerbate pro‐inflammatory SASP factor expression (Figure S1C) (Camell Christina et al. 2021). Here, we found that LPS pre‐treatment of SCs enhanced their killing by senolytics (Figure 1C,D). Neither JAK inhibitor nor LPS changed the senescent markers (Figure S1D,E). Hence, interference with SASP factor expression related to the JAK/STAT pathway decreases the impact of senolytic treatment on human preadipocytes, while “senosensitizing” microenvironmental factors such as those linked to infections increase susceptibility of senolytic‐resistant SCs to senolytic treatment (Tripathi, Nchioua, et al. 2021; Camell Christina et al. 2021).

### 3.2 Senolytics Target the Pro‐Inflammatory/Proapoptotic Subset of Senescent Preadipocytes in Obese Mice

**FIGURE 2:**
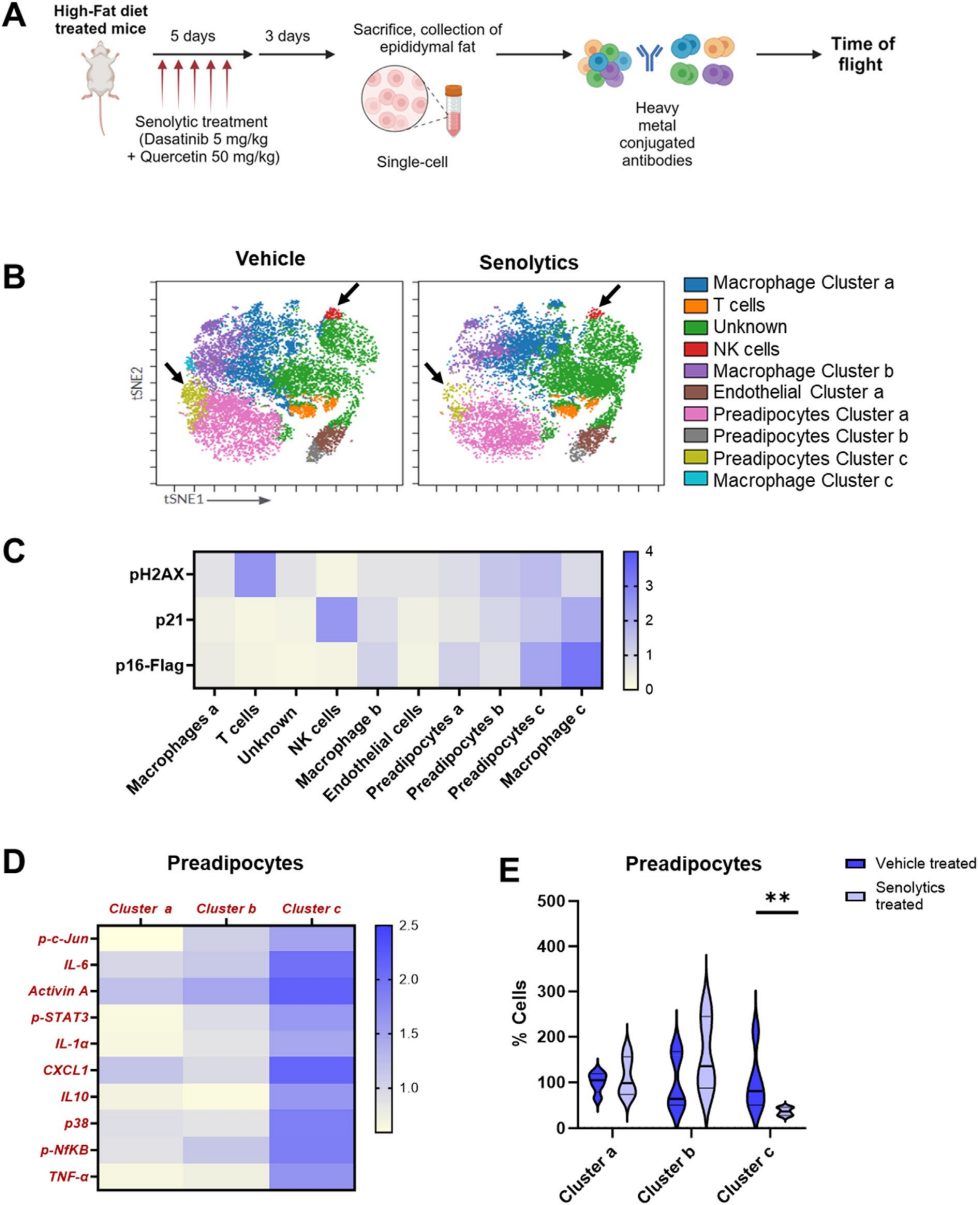
Senolytics target a pro‐inflammatory subset of senescent preadipocytes in obese mice. (A) CyTOF experimental scheme. (B) Representative t‐SNE plots of FlowSOM clusters of cells from obese vehicle‐ or senolytic‐treated mice. (C) Heatmap of senescence marker expression within FlowSOM clusters (*N* = 5). (D) Heatmap comparing expression of pro‐inflammatory SASP factors among preadipocyte clusters in vehicle‐treated obese mice (*N* = 5). (E) Percent of cells cleared in the indicated clusters in senolytic‐ vs. vehicle‐treated mice (*N* = 5). Means ± SEM; unpaired two‐tailed Mann–Whitney tests.

Senolytics were developed based on the observation that those SCs that are tissue‐damaging employ antiapoptotic, pro‐survival pathways (SCAPs) to protect themselves from their own proapoptotic SASP (Zhu et al. 2015). Different senolytics discovered using this hypothesis‐driven approach target 30% to 70% of senescent cells in vitro (Zhu et al. 2015, 2017). Here we tested if it is indeed the senescent cells with high pro‐inflammatory/proapoptotic SASP factor expression that are selectively vulnerable to senolytics in vivo. Fourteen‐ to 16‐month‐old male mice that had been on a high‐fat diet (see Methods) for 8 months were administered Dasatinib (5 mg/kg) plus Quercetin (50 mg/kg) or vehicle (60% Phosal, 10% ethanol, and 30% PEG‐400) for five consecutive days by oral gavage. Mice were euthanized 3 days after the final dose. Organs were harvested and single‐cell suspensions from epididymal fat tissue were analyzed by CyTOF (Figure 2A). Changes in cellular composition caused by senolytics are shown in Figure 2B. Senolytics decreased preadipocyte and immune cell abundance (black arrows). Markers of senescence, including p16^Ink4a^, p21^Cip1^, CENP‐B, and γ‐H2AX, were detected in CD34^+^ cells (preadipocytes) and F4/80^+^ cells (macrophages; Figure 2C). Among preadipocytes, cluster c, a pro‐inflammatory/proapoptotic subset of senescent cells, was decreased by senolytics (Figure 2D,E). With the sample size used in our study, senolytic treatment may have had a slight but yet nonsignificant effect on macrophages, including those that express senescence markers and pro‐inflammatory factors (Figures S2A,B and S3A). Natural killer (NK) cells were reduced (Figure S3A), but these NK cells did not have detectable expression of the DNA damage marker, γ‐H2AX (Figure 2C). Senolytic treatment did not lead to statistically significant changes in endothelial cell abundance, which may relate to their lower expression of senescence markers (Figure 2D). As we reported previously in cultured cells and tissue explants (Xu et al. 2018; Zhu et al. 2015), these findings suggest senolytics primarily target those SCs with a pro‐inflammatory/apoptotic SASP in vivo and that the presence of pro‐inflammatory SCs correlates with immune cell infiltration; however, this needs further investigation.

### 3.3 Markers of Cellular Senescence Do Not Differ Significantly Between the Senolytic‐Resistant and Total SC Populations

**FIGURE 3:**
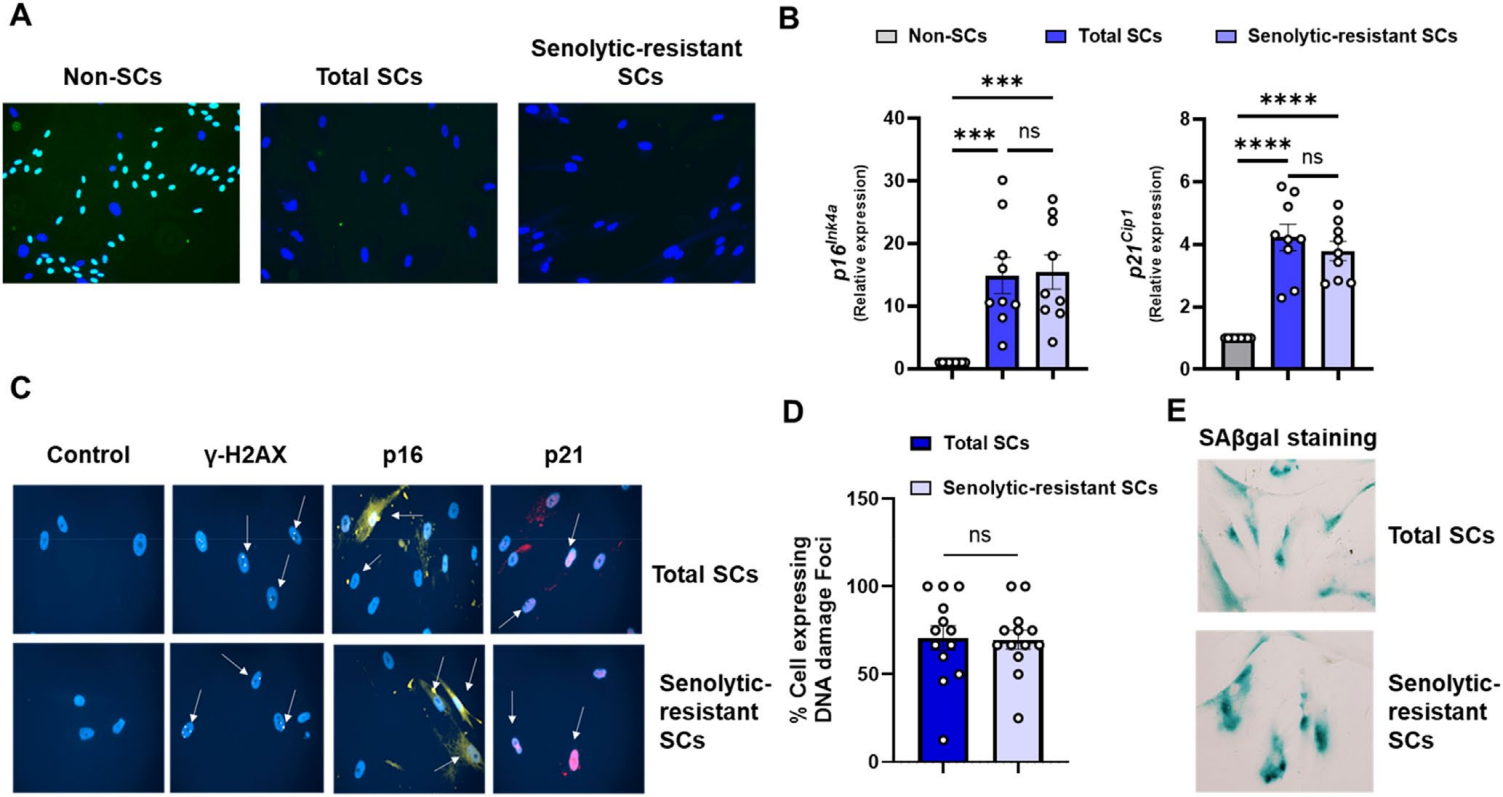
Cellular senescence markers are similar in the human preadipocyte senolytic‐resistant versus total SC preadipocyte populations. (A) Cells were analyzed for proliferative arrest by BrdU staining. See quantification of BrdU positive cells in Figure S4A. (B) Gene expression of the indicated senescence markers (*N* = 9) in total senescent preadipocyte populations vs. senolytic‐resistant senescent preadipocytes. Data are expressed as a function of non‐senescent control cells. Means ± SEM; unpaired, 1‐way ANOVA; *post hoc* comparisons by Tukey's HSD multiple comparison test. (C) Total and senolytic‐resistant preadipocyte SC populations were immunostained for γ‐H2AX, p16^INK4a^, and p21^CIP1^; representative images of *N* = 3 subjects are shown. See quantification of p16^INK4a^, and p21^CIP1^ expression in Figure S4B. (D) Quantification of γ‐H2AX expression. Means ± SEM; unpaired 2‐tailed Student's *t*‐tests. (E) SAβgal (pH 6) in the total and senolytic‐resistant senescent preadipocyte populations. See quantification of SAβgal intensity in Figure S4C. Analogous findings in human senescent endothelial populations are in Figure S4D–F.

To test whether those cells that are resistant to senolytics are indeed senescent, several established markers of senescence were analyzed. We previously showed that the efficacy of different senolytics depends on the cell type of origin (Zhu et al. 2015). For example, Dasatinib targets senescent preadipocytes, while Quercetin targets senescent HUVECs (Zhu et al. 2015). Senescent preadipocytes and HUVECs were treated with Dasatinib or Quercetin, respectively, for 24 h. Next, the remaining cells were washed with PBS 4X and subsequently incubated for 3 days. The resulting senolytic‐resistant SCs were proliferatively arrested as confirmed by BrdU labeling (Figure 3A). Senolytic‐resistant (only cells remaining after exposure to senolytics) were compared to total (senolytic‐resistant plus senolytic‐sensitive) SC populations because it is not yet feasible a priori to isolate only senolytic‐sensitive SCs. In both the total and senolytic‐resistant SC populations, we did not detect statistically significant differences in the markers associated with cell cycle inhibition (p16^INK4a^ and p21^CIP1^) and the DNA damage marker, γ‐H2AX (Figure 3B–D and Figure S4A–C). Like the total SC population, senolytic‐resistant SCs were SAβgal+ (Figure 3E). Furthermore, senescence markers in Quercetin‐treated senescent endothelial cells (HUVECs) were similar to the total senescent HUVEC population (Figure S4D–F). Hence, consistent with our previous reports (Xu et al. 2018; Zhu et al. 2015), we propose, at least in vitro, that SC populations comprise two subsets, the one being susceptible to senolytics and the other resistant.

### 3.4 The SASP of the Senolytic‐Resistant Is Distinct From That of the Total SC Population

**FIGURE 4:**
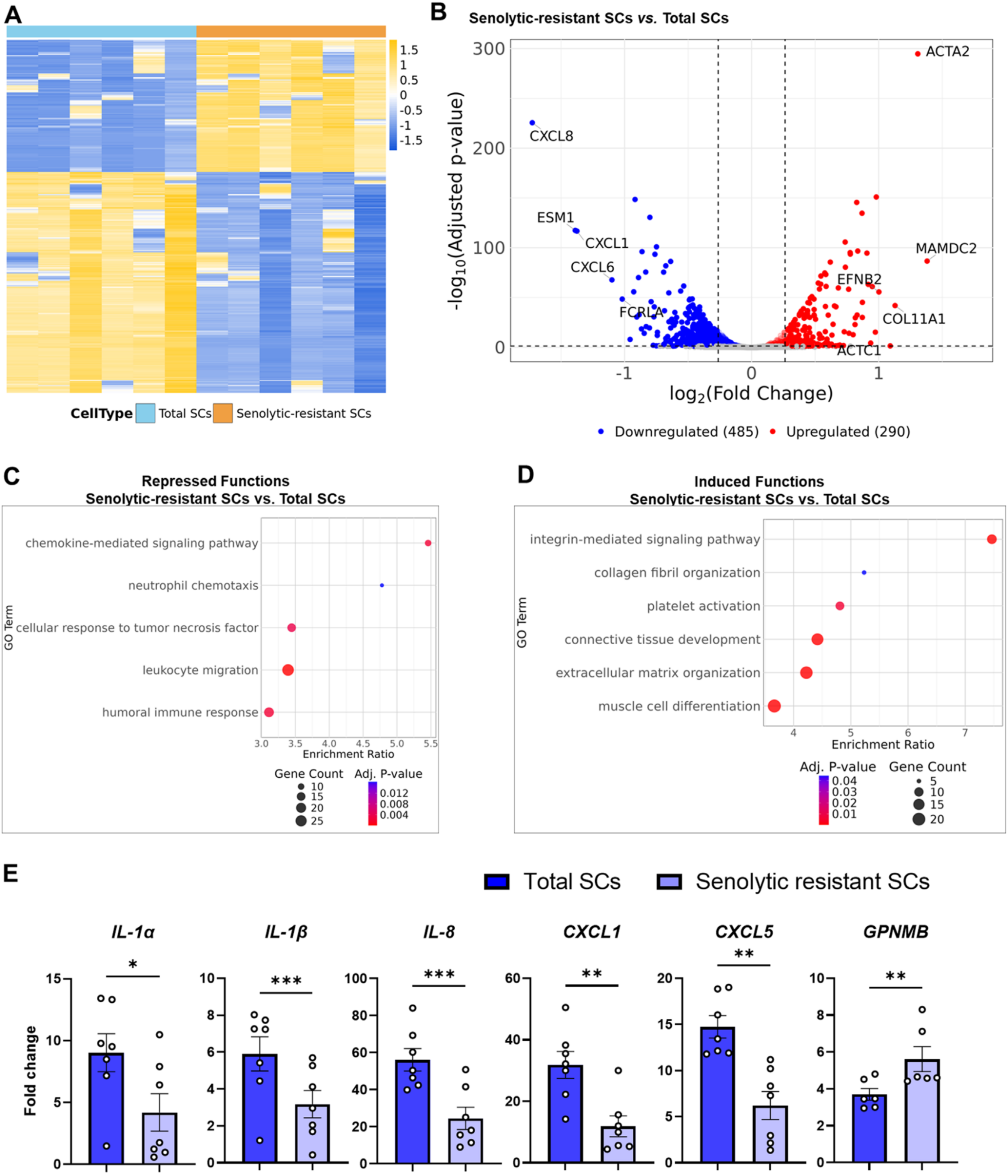
Senolytic‐resistant human senescent preadipocyte SASP profiles are distinct from the total SC population. (A) Heat map of differentially expressed genes (DEGs). (B) Volcano plot of the DEGs indicating key genes. (C, D) Gene ontology analysis of biological functions of DEGs in the total versus senolytic‐resistant SC populations. (E) SASP factor expression (rt‐PCR) in senescent preadipocytes. Data are expressed as a function of vehicle‐treated non‐senescent cells. Means ± SEM; paired, 2‐tailed Student's *t*‐tests.

To test further if senolytic treatment indeed targets pro‐inflammatory senescent subtypes, bulk RNA‐sequencing was conducted of preadipocytes cultured with Dasatinib (senolytic‐resistant SCs) or vehicle (total SCs) from six healthy human kidney transplant donors after inducing senescence by X‐radiation. Clustering of the differential gene expression profiles between senolytic‐resistant versus total SCs is shown in the heat map and volcano plots of key genes (Figure 4A,B). A detailed list of all differentially expressed genes is provided in Table S1. Potential differences in biological function between the senolytic‐resistant versus total human preadipocyte SC populations were ascertained by Gene Ontology over‐representation analysis of the differentially expressed genes (Figure 4C,D, Table S2). Senolytic‐resistant SCs exhibited enrichment of biological processes associated with growth and repair and tissue development compared to the total SC population (Figure 4D). Repressed functions in senolytic‐resistant SCs included activation and migration of immune cells, as further suggested by gene expression and protein analyses (Figure 4C,E, Figure S5), including genes encoding chemokines/cytokines such as *CXCL1*, *CXCL5*, and *CXCL8*. Furthermore, senolytic‐resistant SCs had higher glycoprotein non‐melanoma‐B (GPNMB) expression, a recently identified senescence marker (Saade et al. 2021; Suda et al. 2022, 2021), compared to the total SC population (Figure 4E). GPNMB can impact innate and adaptive immune function, decrease inflammation, promote tumor growth, and shield some cancers from immune/inflammatory defenses (Maric et al. 2013; Lazaratos et al. 2022).

Analogously to preadipocytes, senescent HUVECs resistant to Quercetin had differences in gene expression compared to the total HUVEC SC population, with a less inflamed gene expression pattern in the senolytic‐resistant HUVECs than the total senescent HUVEC population (Figure S6A). With the sample size used in our studies, unlike SCs, non‐senescent control human preadipocyte or HUVEC cultures exposed to senolytics did not have statistically significant reductions in their already low pro‐inflammatory SASP factor expression compared to cells exposed to vehicle (Figure S6B,C). Although some SASP factors were upregulated in non‐senescent cells treated with senolytics, the levels of these pro‐inflammatory factors remained lower than SCs. This observation suggests that while senolytics clear SCs and reduce inflammation over the long term, they can also elicit stress adaptation responses by short‐term, transient activation of pro‐inflammatory signals in non‐senescent cells. These findings support the possibility that senolytics primarily target the SC subtype with an inflammatory, apoptosis‐inducing SASP profile, consistent with the hypothesis‐driven approach that we used to discover the first senolytics (Zhu et al. 2015).

### 3.5 Senolytic‐Resistant Human Senescent Preadipocytes Differ in Extent of Induction of Inflammation and mt‐DNA Secretion From the Total SC Population

**FIGURE 5:**
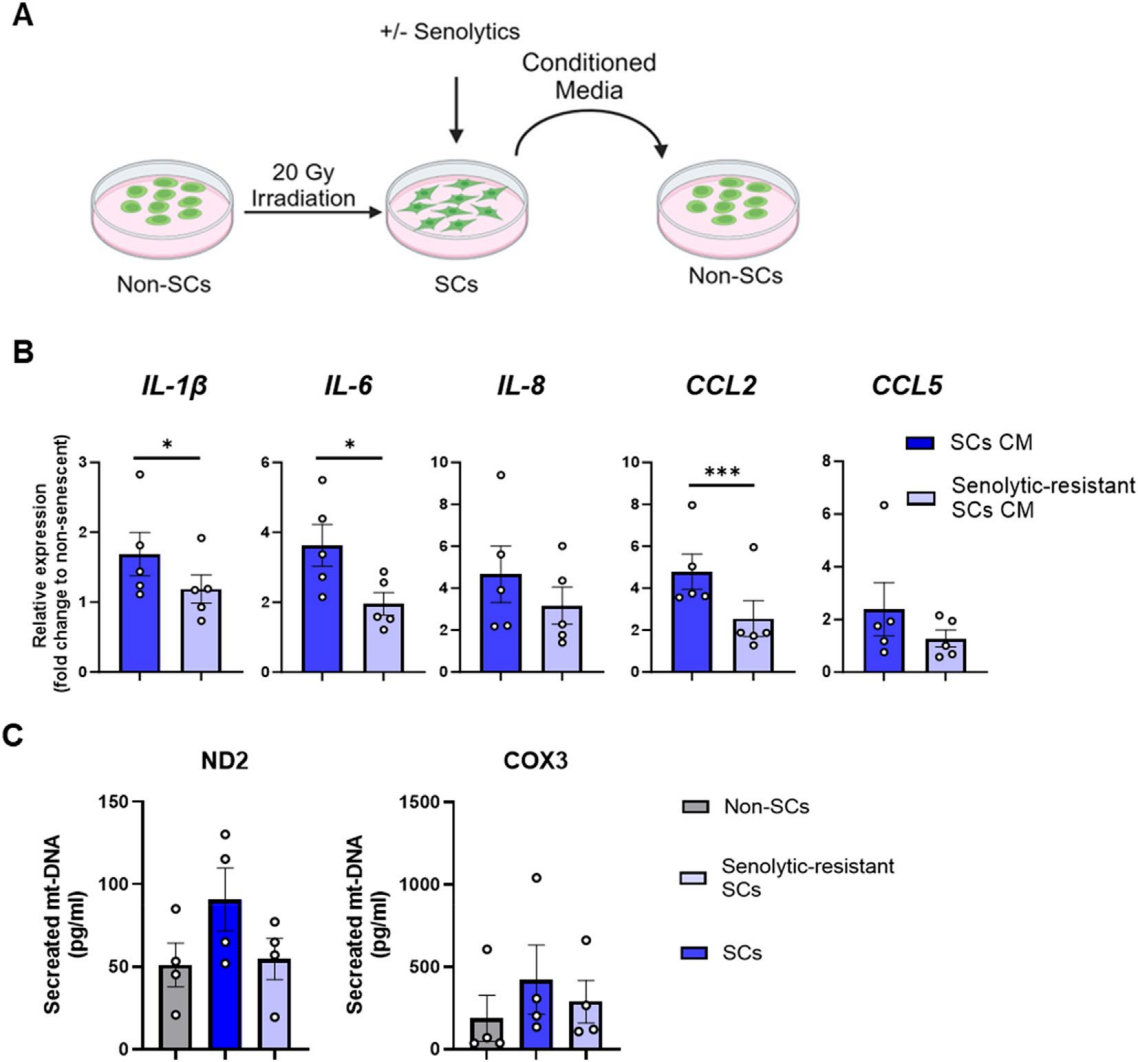
Effects of the senolytic‐sensitive vs. total SC populations on induction of inflammation and secretion of mt‐DNA. (A) Experimental scheme. (B) Non‐senescent preadipocytes were treated with conditioned media (CM) from resistant vs. total SC populations for 24 h, and inflammatory factors were analyzed by rt‐PCR. Means ± SEM; paired, two‐tailed Student's *t*‐tests. (C) Secreted mt‐DNA by indicated cell types. Means ± SEM; paired, 1‐way ANOVA; post hoc pairwise comparison by Tukey's HSD multiple comparison test.

Senescent preadipocytes can spread an inflammatory state to non‐senescent cells and activate immune cells, in part through SC mtDNA production and release (Xu et al. 2015; Iske et al. 2020). This induction of inflammation by SC mtDNA was recently confirmed (Zhu et al. 2017; Victorelli et al. 2023). Given the differences in SASP profiles between senolytic‐resistant and the total SC populations, whether the SC subtypes induce inflammation to the same extent in non‐senescent cells was tested (Figure 5A). Non‐senescent preadipocytes from healthy donors had lower expression of inflammatory factors when exposed to CM from senolytic‐resistant cells compared to those cultured with CM prepared from the total SC population (Figure 5B). Furthermore, cell‐free mtDNA content in CM, marked by expression of NADH dehydrogenase subunit 2 (MT‐ND2) and cytochrome c oxidase subunit III (MT‐COX3) of the electron transport chain (known to be proapoptotic (Iske et al. 2020; Victorelli et al. 2023)) tended to be higher in cultures of the total SC population than in cultures of the senolytic‐resistant SC population (Figure 5C), unlike cell‐free nuclear DNA (Figure S7). Hence, senolytic‐resistant SCs appear to provoke less inflammation than the total population of SCs (senolytic‐resistant plus senolytic‐sensitive SCs).

### 3.6 Transplanting Senolytic‐Resistant Subtype SCs Causes Less Physical Dysfunction Than Transplanting the Total SC Population

**FIGURE 6:**
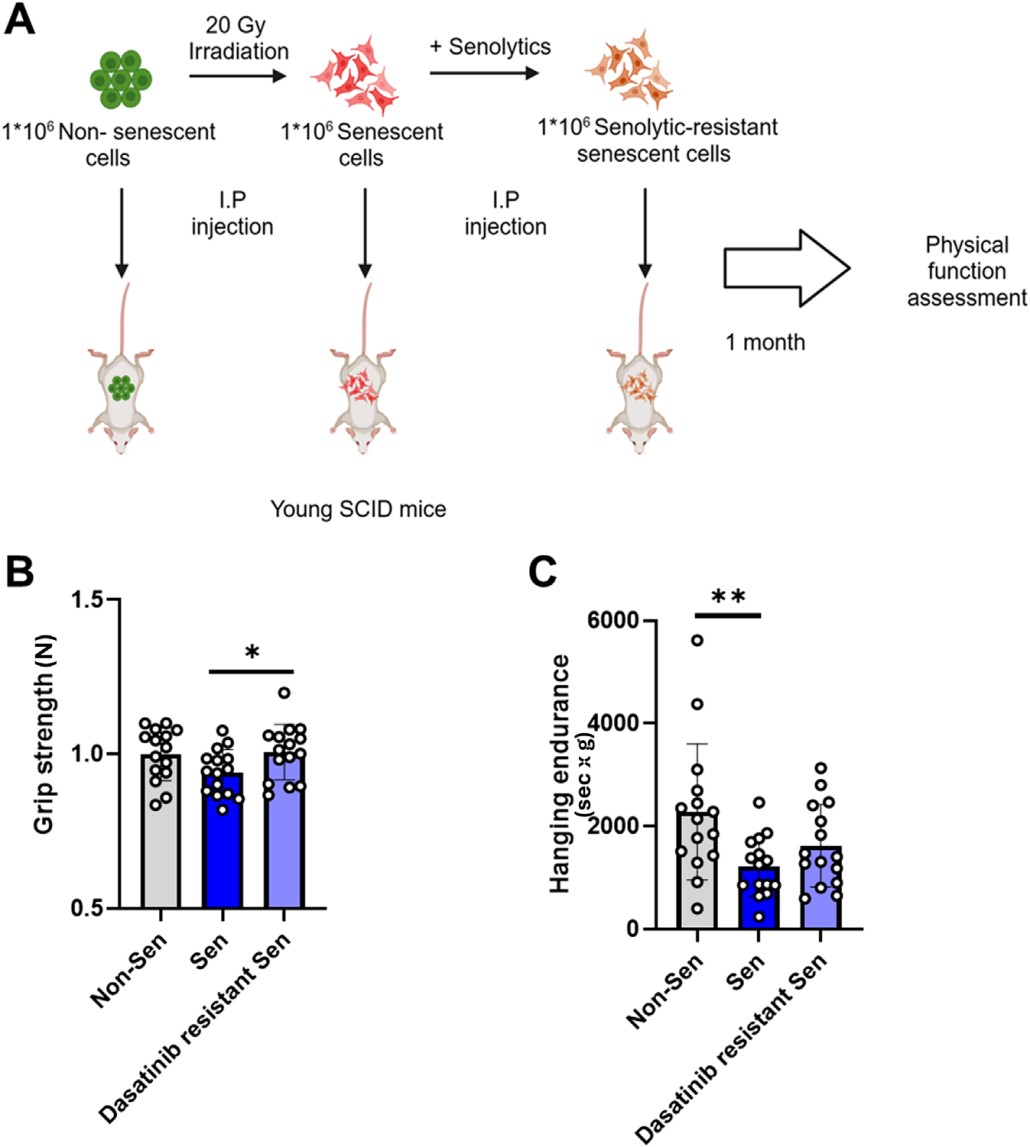
SC subtypes transplanted into younger mice differ in impact on physical function. (A) Experimental scheme. (B) Grip strength and (C) wire hanging endurance (sec × BW g) in 2‐month‐old male *SCID‐Beige* mice 1 month after being transplanted with 1 × 10^6^ senolytic‐resistant or total senescent human preadipocyte population cells by intraperitoneal injection (*N* = 15). Baseline grip strength was similar in each group of mice before transplantation (Figure S9). Data are shown as means ± SEM with individual values; unpaired two‐tailed Student's *t*‐tests.

Transplanting small numbers of SCs into mice is sufficient to induce features of frailty, suggesting a role of SCs in causing physical dysfunction (Xu et al. 2018). To test whether the SC subtypes differ in their impact on physical function in vivo, 1 × 10^6^ senolytic‐resistant or total population SCs were transplanted intraperitoneally into 2‐month‐old male young *SCID‐Beige* mice (Figure 6A). After 1 month, physical function variables were tested. In previously healthy mice that were transplanted with total SC population cells, reductions in grip strength (Figure 6B) and hanging endurance (Figure 6C) were more evident than in mice transplanted with senolytic‐resistant SCs, indicating differences between the impacts of the SC subtypes on function.

### 3.7 A “1:2:3:4”‐Step Approach Enhances Ablation of Cultured Triple‐Negative Breast Cancer Cells

Lastly, we explored the potential application of senosensitizers in the context of cancer. Triple‐negative breast cancer (TNBC) remains one of the most challenging malignancies to treat due to its poor prognosis and resistance to conventional therapies. It has been found that chemotherapy can induce a senescent phenotype in cancer cells and that senolytics can eliminate some of these therapy‐induced senescent (TIS) cells (Pacifico et al. 2024). Based on these points and our findings above about senolytic‐sensitive versus‐resistant SCs, we hypothesized senosensitizers could enhance the effectiveness of senolytics in removing TIS. We previously found TLR3 agonists (which activate the PAMP pathway also activated by coronaviruses) upregulate pro‐inflammatory/proapoptotic SASP factors in senescent cells (Tripathi, Nchioua, et al. 2021). Since LPS is not likely to become a feasible senosensitizing intervention in humans, we used a TLR3 agonist as a senosensitizer. As a preliminary test of our hypothesis, we treated human TNBC MDA‐MB‐231 cells with Cisplatin, followed by Dasatinib, then the TLR3 agonist polyinosinic‐polycytidylic as a senosensitizer, and finally Dasatinib again (Figure S9). This “1:2:3:4” stepwise regimen (chemotherapy → senolytic → senosensitizer → senolytic) resulted in a ~90% further reduction in TNBC cell survival than cells treated with chemotherapy alone (Figure S9).

## 4 Discussion

Based on differences in SASP profiles, responsiveness to senolytics, and functional outcomes, we propose a conceptual framework distinguishing senolytic‐sensitive and senolytic‐resistant senescent cell subtypes. While these categories are not absolute or mutually exclusive, they provide a useful lens for interpreting observed heterogeneity in senescent cell behavior. Both have characteristics of SCs (increased p16^INK4a^, p21^CIP1^, γH2AX, SAβgal, and proliferative arrest), but the subtypes differ in SASP factors produced, mtDNA release, responsiveness to senolytics, and functional effects in vitro and in vivo. These “senolytic‐sensitive” and “senolytic‐resistant” SC subtypes are reminiscent of the “deleterious” and “helper” SC subtypes previously proposed (Tripathi, Misra, et al. 2021). The subpopulations might arise in response to pre‐existing diversity within non‐senescent cells, with effects being amplified once cells have become senescent through such mechanisms as: (1) expression of genes that had been silent in non‐senescent cells due to epigenetic changes and associated chromatin openness in SCs, transpositional events, or other SC‐intrinsic mechanisms, (2) differences in mtDNA abundance or localization between the senolytic‐resistant and ‐sensitive SC subtypes (mtDNA is linked to inflammation in SCs (Iske et al. 2020)), (3) host *milieu*, including characteristics of cells near the SCs (Teo et al. 2019), or (4) extent of replication of cells before they originally became senescent, among other potential mechanisms or combinations of mechanisms. The senolytic‐resistant SC subtype appears to have a more strongly pro‐repair, fibrotic, and growth promoting profile, possibly in part related to TGF‐β (Grande 1997), than senolytic‐sensitive SCs. The senolytic‐sensitive subtype has a SASP that is inflammatory, with higher expression of cytokines and chemokines and more mtDNA release than the senolytic‐resistant subtype. In the present study, cells exposed to conditioned media from the total population of SCs exhibited more pronounced inflammatory features than those treated with media from senolytic‐resistant SCs. In both groups we couldn't observe the difference in senescence markers in this study.

The first senolytics were discovered based on the hypothesis that SCs resist apoptotic stimuli related to increased pro‐survival, antiapoptotic defenses and the finding that targeting these SC anti‐apoptotic pathways leads to elimination of 30%–70% of SCs (Zhu et al. 2015). Consistent with this, the SASP of senolytic‐resistant SCs is less pro‐inflammatory, proapoptotic, and tissue destructive than the SASP of senolytic‐sensitive SCs, although in future studies, this conclusion would be further strengthened by analysis of secreted protein levels rather than gene expression alone. Additionally, suppressing or amplifying pro‐inflammatory SASP features, respectively, decreased or enhanced extent of SC killing by senolytics. The interplay between the SASP and SCAPs and verification that Ruxolitinib or LPS modulated apoptosis (caspase assays, TUNEL) need to be explored in future studies. Single‐cell proteomic studies in mice with obesity, a state marked by increased pro‐inflammatory SC abundance especially in epididymal fat depots, indicated that within the senescent preadipocyte population (marked by p16+, p21^Cip1^+, γH2AX+), a cluster of cells highly expressing pro‐inflammatory/proapoptotic factors was more effectively eliminated by senolytics than other cell populations. Furthermore, SCs can amplify inflammatory factor production in non‐senescent cells (Xu et al. 2015) and here we found that it is principally the subset of senolytic‐sensitive SCs that does so.

Infiltration of immune cells into adipose tissue together with increased SC abundance is a feature of both aging and obesity (Xu et al. 2015). In the t‐SNE analysis, several immune cell clusters, including those corresponding to NK cells and macrophages, appeared to be reduced following senolytic treatment in obese mice. However, only the NK cell population, which is an innate immune cell type that can eliminate persisting SCs (Krizhanovsky et al. 2008), exhibited a statistically significant decrease. This is reminiscent of the observation that migration of tail vein‐injected labeled monocytes into adipose tissue of obese mice treated with senolytics is less than that in obese mice treated with vehicle (Palmer et al. 2019). Hence, senolytic‐induced decreases in the burden of senolytic‐sensitive SCs that produce inflammatory factors and chemokines may reduce immune cell infiltration and activation in vivo, although further exploration is needed to determine if NK cells were eliminated by senolytics or were recruited to a lower extent due to reduced inflammation. The reduced immune infiltration may occur despite the continued presence of senolytic‐resistant SCs because these cells are less pro‐inflammatory and may express more GPMNB than non‐senescent cells, possibilities that merit further investigation.

We investigated the Dasatinib‐ and Quercetin‐resistant versus‐sensitive human preadipocyte and endothelial SC subtypes, respectively. Potentially, other cell types becoming senescent or other senolytics could result in senolytic‐resistant SC subsets with characteristics fundamentally distinct from those studied here. However, in keeping with findings in these two human cell types, a recent study indicated that a subset of senescent fibroblasts with a pro‐inflammatory SASP profile related to NF‐κB and the YAP‐TEAD complex is more susceptible to the senolytic, VPF, than other senescent fibroblast subsets (Anerillas et al. 2023). Further studies are needed to ascertain whether the senolytic‐sensitive versus‐resistant SC subtypes with their apoptotic, tissue‐damaging versus pro‐growth profiles, respectively, occur across more cell types and over a range of different senolytics.

Senolytic‐resistant SCs could have detrimental effects. PAMPs generated during infections may rapidly alter the SASP of pre‐existing senolytic‐resistant SCs, with substantially increased release of tissue‐damaging, inflammatory, proapoptotic factors, potentially worsening symptoms and contributing to complications, perhaps including cytokine storm. Indeed, LPS can amplify inflammatory SASP factors in SCs by over an order of magnitude within 3 h; hence, effects of pathogenic infections on senolytic‐resistant cells need to be examined (Camell Christina et al. 2021). In malignancies, therapy‐induced senolytic‐resistant SCs may produce growth factors that promote cancer growth, express immune evasion signals such as GPNMB, and release pro‐fibrotic factors that create a matrix shielding cancers from immune clearance, speculations that also need to be explored. Senolytic‐resistant cancer therapy (radiation or chemotherapy)‐induced SCs (TIS) harboring cancerous mutations could lead to tumor relapse once these SCs have accumulated further mutations and escape senescence (Kirkland 2023). Therefore, there may be situations in which strategies for ablating senolytic‐resistant “helper” SCs may be important over and above eliminating only proapoptotic/pro‐inflammatory SCs (Figure S11).

Studies are needed to test whether these hypotheses are correct, because an asymptomatic reservoir of “helper”, senolytic‐resistant SCs could build up with aging or after such senescence‐inducing insults as trauma, obesity/diabetes, chemotherapy, radiation, or infections, among others. To this end, because proapoptotic, inflammatory SASP features can be attenuated by senomorphics (e.g., Ruxolitinib [Figure 1], Metformin (Moiseeva et al. 2013), or Rapamycin (Laberge et al. 2015)) and be rapidly upregulated by PAMPs (e.g., LPS or TLR3 activation in response to coronavirus (Camell Christina et al. 2021); (Tripathi, Nchioua, et al. 2021)), we speculate that the impact of senolytics might be reduced by senomorphics and enhanced by agents related to PAMPs (Figure S11). If senomorphics do indeed impede effectiveness of senolytics, this would imply that simultaneous (as opposed to sequential) administration of senolytics and senomorphics may have less than additive effects in delaying, preventing, alleviating, or treating senescence‐related disorders and diseases. Conversely, accentuating apoptotic SASP factor production by exposing senolytic‐resistant SCs to agents currently under investigation, “senosensitizers”, which act through PAMP‐related mechanisms, may sensitize previously senolytic‐resistant SCs to senolytics. Potentially, this might defend against complications of insults such as infections in individuals harboring previously clinically silent senolytic‐resistant SCs. In patients with malignancies undergoing chemotherapy/radiation, we also speculate that administration of senosensitizers could boost the numbers of cancer‐harboring TIS that can be removed subsequently by senolytics (Figure S10). In this scenario, effectiveness of standard chemotherapy/radiation could possibly be augmented by a “1‐2‐3‐4 punch” approach: (1) Chemotherapy/radiation; (2) A round of senolytics to remove senolytic‐sensitive TIS; (3) A round of senosensitizers to enhance responsiveness to senolytics of any remaining cancer‐harboring, previously senolytic‐resistant TIS; and (4) An additional round of senolytics to remove these now senolytic‐sensitive remaining cells. Early data supporting hypothesis in the case of triple negative breast cancer are shown in Figure S10. Hence, there is a pressing need to comprehensively characterize senolytic‐resistant SCs and develop strategies to eliminate these cells, including developing effective senosensitizers and therapeutic regimens entailing sequencing administration of senosensitizers and senolytics. Although the data presented in this manuscript are based on a limited number of subjects and cell types, which may raise concerns regarding generalizability, they have the potential to establish a new paradigm in cancer and senescence‐targeted therapies. In our view, these provocative findings warrant further investigation.

## Author Contributions

U.T., T.T., and J.L.K. generated the overall concept of the study. U.T., M.S., T.T., and J.L.K. designed the study and wrote the manuscript. U.T., M.S., C.I., A.P., V.K., K.J., T.P., S.C., L.P.G.L.P., and Y.Z. performed experiments. B.T.P., H.K.Y., and R.M. analyzed the RNA‐seq data. N.G. and M.X. assisted with RNA‐seq. N.G. conducted the isolation of preadipocytes from human fat tissues. D.B.A., H.K.Y., and R.M. supervised the statistical analyses. All authors read, edited, and approved the final version of the manuscript.

## Funding

This work was supported by NIH grants R37AG013925 (J.L.K., T.T.), R33AG061456 (J.L.K., T.T.), R01AG066679 (M.X.), R01AG076642 (M.X.), R01AG064165 (S.G.T.), R01AG087387 (Y.Z.), the Connor Fund (J.L.K., T.T.), Robert J. and Theresa W. Ryan (J.L.K., T.T.), HF‐GRO‐23‐1199148‐3 (J.L.K.), HF‐GRO‐23‐1199262‐27 (Y.Z., M.S.), the JSPS Grants‐in‐Aid for Scientific Research Fund for the Promotion of Joint International Research (Fostering Joint International Research) 23KK0295 (M.S.), the Yamada Science Foundation (M.S.), and USDA/ARS grant CRIS 3092‐51000‐065‐003S (H.K.Y.) and the Noaber Foundation (J.L.K.). U.T. was supported by an American Heart Association predoctoral fellowship (917775).

## Conflicts of Interest

Patents and pending patents about senolytic drugs and senosensitizers and their uses are held by Mayo Clinic. This research was reviewed by the Mayo Clinic Conflicts of Interest Review Board and conducted in compliance with Mayo Clinic and Cedars‐Sinai policies.

## Supporting information


**Appendix S1:** acel70358‐sup‐0001‐AppendixS1.zip.

## Data Availability

All raw data used to generate graphs are included in Appendix S1. Raw sequencing data and gene counts are available through NCBI's Gene Expression repository under accession number: GSE268701.

## References

[acel70358-bib-0001] Anerillas, C. , K. Mazan‐Mamczarz , A. B. Herman , et al. 2023. “The YAP–TEAD Complex Promotes Senescent Cell Survival by Lowering Endoplasmic Reticulum Stress.” Nature Aging 3, no. 10: 1237–1250. 37667102 10.1038/s43587-023-00480-4 PMC11369890

[acel70358-bib-0002] Baker, D. J. , T. Wijshake , T. Tchkonia , et al. 2011. “Clearance of p16Ink4a‐Positive Senescent Cells Delays Ageing‐Associated Disorders.” Nature 479, no. 7372: 232–236. 22048312 10.1038/nature10600 PMC3468323

[acel70358-bib-0003] Benjamini, Y. , and Y. Hochberg . 1995. “Controlling the False Discovery Rate: A Practical and Powerful Approach to Multiple Testing.” Journal of the Royal Statistical Society. Series B, Statistical Methodology 57, no. 1: 289–300.

[acel70358-bib-0004] Bolger, A. M. , M. Lohse , and B. Usadel . 2014. “Trimmomatic: A Flexible Trimmer for Illumina Sequence Data.” Bioinformatics 30, no. 15: 2114–2120. 24695404 10.1093/bioinformatics/btu170 PMC4103590

[acel70358-bib-0005] Born, E. , L. Lipskaia , M. Breau , et al. 2023. “Eliminating Senescent Cells Can Promote Pulmonary Hypertension Development and Progression.” Circulation 147, no. 8: 650–666. 36515093 10.1161/CIRCULATIONAHA.122.058794

[acel70358-bib-0006] Camell Christina, D. , M. J. Yousefzadeh , Y. Zhu , et al. 2021. “Senolytics Reduce Coronavirus‐Related Mortality in Old Mice.” Science 373, no. 6552: eabe4832. 34103349 10.1126/science.abe4832 PMC8607935

[acel70358-bib-0007] Campisi, J. 2005. “Senescent Cells, Tumor Suppression, and Organismal Aging: Good Citizens, Bad Neighbors.” Cell 120, no. 4: 513–522. 15734683 10.1016/j.cell.2005.02.003

[acel70358-bib-0008] Chaib, S. , J. A. López‐Domínguez , M. Lalinde‐Gutiérrez , et al. 2024. “The Efficacy of Chemotherapy Is Limited by Intratumoral Senescent Cells Expressing PD‐L2.” Nature Cancer 5, no. 3: 448–462. 38267628 10.1038/s43018-023-00712-x PMC10965441

[acel70358-bib-0009] Cubro, H. , K. A. Nath , S. Suvakov , et al. 2021. “Mechanisms of Vascular Dysfunction in the Interleukin‐10‐Deficient Murine Model of Preeclampsia Indicate Nitric Oxide Dysregulation.” Kidney International 99, no. 3: 646–656. 33144212 10.1016/j.kint.2020.09.034 PMC7914163

[acel70358-bib-0010] De Cecco, M. , T. Ito , A. P. Petrashen , et al. 2019. “L1 Drives IFN in Senescent Cells and Promotes Age‐Associated Inflammation.” Nature 566, no. 7742: 73–78. 30728521 10.1038/s41586-018-0784-9 PMC6519963

[acel70358-bib-0011] Demaria, M. 2014. “An Essential Role for Senescent Cells in Optimal Wound Healing Through Secretion of PDGF‐AA.” Developmental Cell 31: 722–733. 25499914 10.1016/j.devcel.2014.11.012 PMC4349629

[acel70358-bib-0012] Farr, J. N. , M. Xu , M. M. Weivoda , et al. 2017. “Targeting Cellular Senescence Prevents Age‐Related Bone Loss in Mice.” Nature Medicine 23, no. 9: 1072–1079. 10.1038/nm.4385 PMC5657592 28825716

[acel70358-bib-0013] Gasek, N. S. , G. A. Kuchel , J. L. Kirkland , and M. Xu . 2021. “Strategies for Targeting Senescent Cells in Human Disease.” Nature Aging 1, no. 10: 870–879. 34841261 10.1038/s43587-021-00121-8 PMC8612694

[acel70358-bib-0014] Gorbunova, V. , A. Seluanov , P. Mita , et al. 2021. “The Role of Retrotransposable Elements in Ageing and Age‐Associated Diseases.” Nature 596, no. 7870: 43–53. 34349292 10.1038/s41586-021-03542-y PMC8600649

[acel70358-bib-0015] Grande, J. P. 1997. “Role of Transforming Growth Factor‐Beta in Tissue Injury and Repair.” Proceedings of the Society for Experimental Biology and Medicine 214, no. 1: 27–40. 9012358 10.3181/00379727-214-44066

[acel70358-bib-0016] He, S. , and N. E. Sharpless . 2017. “Senescence in Health and Disease.” Cell 169, no. 6: 1000–1011. 28575665 10.1016/j.cell.2017.05.015 PMC5643029

[acel70358-bib-0017] Iske, J. , M. Seyda , T. Heinbokel , et al. 2020. “Senolytics Prevent Mt‐DNA‐Induced Inflammation and Promote the Survival of Aged Organs Following Transplantation.” Nature Communications 11, no. 1: 4289. 10.1038/s41467-020-18039-x PMC7453018 32855397

[acel70358-bib-0018] Kale, A. , A. Sharma , A. Stolzing , P. Y. Desprez , and J. Campisi . 2020. “Role of Immune Cells in the Removal of Deleterious Senescent Cells.” Immunity & Ageing 17, no. 1: 16. 32518575 10.1186/s12979-020-00187-9 PMC7271494

[acel70358-bib-0019] Katsuumi, G. , I. Shimizu , M. Suda , et al. 2024. “SGLT2 Inhibition Eliminates Senescent Cells and Alleviates Pathological Aging.” Nature Aging 4, no. 7: 926–938. 38816549 10.1038/s43587-024-00642-y PMC11257941

[acel70358-bib-0020] Khosla, S. , J. N. Farr , T. Tchkonia , and J. L. Kirkland . 2020. “The Role of Cellular Senescence in Ageing and Endocrine Disease.” Nature Reviews. Endocrinology 16, no. 5: 263–275. 10.1038/s41574-020-0335-y PMC7227781 32161396

[acel70358-bib-0021] Kim, D. , B. Langmead , and S. L. Salzberg . 2015. “HISAT: A Fast Spliced Aligner With Low Memory Requirements.” Nature Methods 12, no. 4: 357–360. 25751142 10.1038/nmeth.3317 PMC4655817

[acel70358-bib-0022] Kirkland, J. L. 2023. “Tumor Dormancy and Disease Recurrence.” Cancer Metastasis Reviews 42, no. 1: 9–12. 36877312 10.1007/s10555-023-10096-0 PMC9986652

[acel70358-bib-0023] Kirkland, J. L. , and T. Tchkonia . 2020. “Senolytic Drugs: From Discovery to Translation.” Journal of Internal Medicine 288, no. 5: 518–536. 32686219 10.1111/joim.13141 PMC7405395

[acel70358-bib-0024] Krizhanovsky, V. , M. Yon , R. A. Dickins , et al. 2008. “Senescence of Activated Stellate Cells Limits Liver Fibrosis.” Cell 134, no. 4: 657–667. 18724938 10.1016/j.cell.2008.06.049 PMC3073300

[acel70358-bib-0025] Laberge, R.‐M. , Y. Sun , A. V. Orjalo , et al. 2015. “MTOR Regulates the Pro‐Tumorigenic Senescence‐Associated Secretory Phenotype by Promoting IL1A Translation.” Nature Cell Biology 17, no. 8: 1049–1061. 26147250 10.1038/ncb3195 PMC4691706

[acel70358-bib-0026] Lazaratos, A.‐M. , M. G. Annis , and P. M. Siegel . 2022. “GPNMB: A Potent Inducer of Immunosuppression in Cancer.” Oncogene 41, no. 41: 4573–4590. 36050467 10.1038/s41388-022-02443-2

[acel70358-bib-0027] Maric, G. , A. A. Rose , M. G. Annis , and P. M. Siegel . 2013. “Glycoprotein Non‐Metastatic b (GPNMB): A Metastatic Mediator and Emerging Therapeutic Target in Cancer.” Oncotargets and Therapy 6: 839–852. 23874106 10.2147/OTT.S44906 PMC3711880

[acel70358-bib-0028] Moiseeva, O. , X. Deschênes‐Simard , E. St‐Germain , et al. 2013. “Metformin Inhibits the Senescence‐Associated Secretory Phenotype by Interfering With IKK/NF‐κB Activation.” Aging Cell 12, no. 3: 489–498. 23521863 10.1111/acel.12075

[acel70358-bib-0029] Moncsek, A. , M. S. al‐Suraih , C. E. Trussoni , et al. 2018. “Targeting Senescent Cholangiocytes and Activated Fibroblasts With B‐Cell Lymphoma‐Extra Large Inhibitors Ameliorates Fibrosis in Multidrug Resistance 2 Gene Knockout (Mdr2(−/−)) Mice.” Hepatology 67, no. 1: 247–259. 28802066 10.1002/hep.29464 PMC5739965

[acel70358-bib-0030] Muñoz‐Espín, D. , and M. Serrano . 2014. “Cellular Senescence: From Physiology to Pathology.” Nature Reviews Molecular Cell Biology 15, no. 7: 482–496. 24954210 10.1038/nrm3823

[acel70358-bib-0031] Ogrodnik, M. , Y. Zhu , L. G. P. Langhi , et al. 2019. “Obesity‐Induced Cellular Senescence Drives Anxiety and Impairs Neurogenesis.” Cell Metabolism 29, no. 5: 1061–1077.e8. 30612898 10.1016/j.cmet.2018.12.008 PMC6509403

[acel70358-bib-0032] Pacifico, F. , F. Magni , A. Leonardi , and E. Crescenzi . 2024. “Therapy‐Induced Senescence: Novel Approaches for Markers Identification.” International Journal of Molecular Sciences 25, no. 15: 8448. 39126015 10.3390/ijms25158448 PMC11313450

[acel70358-bib-0033] Palmer, A. K. , T. Tchkonia , and J. L. Kirkland . 2022. “Targeting Cellular Senescence in Metabolic Disease.” Molecular Metabolism 66: 101601. 36116755 10.1016/j.molmet.2022.101601 PMC9520013

[acel70358-bib-0034] Palmer, A. K. , M. Xu , Y. Zhu , et al. 2019. “Targeting Senescent Cells Alleviates Obesity‐Induced Metabolic Dysfunction.” Aging Cell 18, no. 3: e12950. 30907060 10.1111/acel.12950 PMC6516193

[acel70358-bib-0035] Prata, L. , L. G. P. L. Prata , I. G. Ovsyannikova , T. Tchkonia , and J. L. Kirkland . 2018. “Senescent Cell Clearance by the Immune System: Emerging Therapeutic Opportunities.” Seminars in Immunology 40: 101275. 31088710 10.1016/j.smim.2019.04.003 PMC7061456

[acel70358-bib-0036] Robinson, M. D. , D. J. McCarthy , and G. K. Smyth . 2009. “edgeR: A Bioconductor Package for Differential Expression Analysis of Digital Gene Expression Data.” Bioinformatics 26, no. 1: 139–140. 19910308 10.1093/bioinformatics/btp616 PMC2796818

[acel70358-bib-0037] Roos, C. M. , B. Zhang , A. K. Palmer , et al. 2016. “Chronic Senolytic Treatment Alleviates Established Vasomotor Dysfunction in Aged or Atherosclerotic Mice.” Aging Cell 15, no. 5: 973–977. 26864908 10.1111/acel.12458 PMC5013022

[acel70358-bib-0038] Saade, M. , G. Araujo de Souza , C. Scavone , and P. F. Kinoshita . 2021. “The Role of GPNMB in Inflammation.” Frontiers in Immunology 12: 674739. 34054862 10.3389/fimmu.2021.674739 PMC8149902

[acel70358-bib-0039] Saccon, T. D. , R. Nagpal , H. Yadav , et al. 2021. “Senolytic Combination of Dasatinib and Quercetin Alleviates Intestinal Senescence and Inflammation and Modulates the Gut Microbiome in Aged Mice.” Journals of Gerontology. Series A, Biological Sciences and Medical Sciences 76, no. 11: 1895–1905. 33406219 10.1093/gerona/glab002 PMC8514064

[acel70358-bib-0040] Schafer, M. J. , T. A. White , K. Iijima , et al. 2017. “Cellular Senescence Mediates Fibrotic Pulmonary Disease.” Nature Communications 8: 14532. 10.1038/ncomms14532 PMC5331226 28230051

[acel70358-bib-0041] Stout, M. B. , T. Tchkonia , T. Pirtskhalava , et al. 2014. “Growth Hormone Action Predicts Age‐Related White Adipose Tissue Dysfunction and Senescent Cell Burden in Mice.” Aging (Albany NY) 6, no. 7: 575–586. 25063774 10.18632/aging.100681 PMC4153624

[acel70358-bib-0042] Suda, M. , S. Chaib , L. G. P. Langhi Prata , et al. 2025. “Endothelial Senescent‐Cell‐Specific Clearance Alleviates Metabolic Dysfunction in Obese Mice.” Cell Metabolism 37: 2455–2465.e6. 41270738 10.1016/j.cmet.2025.10.009 PMC12981281

[acel70358-bib-0043] Suda, M. , G. Katsuumi , T. Tchkonia , et al. 2023. “Potential Clinical Implications of Senotherapies for Cardiovascular Disease.” Circulation Journal 88: 277–284. 37880106 10.1253/circj.CJ-23-0657 PMC10922738

[acel70358-bib-0044] Suda, M. , K. H. Paul , T. Minamino , et al. 2023. “Senescent Cells: A Therapeutic Target in Cardiovascular Diseases.” Cells 12, no. 9: 1296. 37174697 10.3390/cells12091296 PMC10177324

[acel70358-bib-0045] Suda, M. , K. H. Paul , U. Tripathi , T. Minamino , T. Tchkonia , and J. L. Kirkland . 2024. “Targeting Cell Senescence and Senolytics: Novel Interventions for Age‐Related Endocrine Dysfunction.” Endocrine Reviews 45, no. 5: 655–675. 38500373 10.1210/endrev/bnae010 PMC11405506

[acel70358-bib-0046] Suda, M. , I. Shimizu , G. Katsuumi , et al. 2022. “Glycoprotein Nonmetastatic Melanoma Protein B Regulates Lysosomal Integrity and Lifespan of Senescent Cells.” Scientific Reports 12, no. 1: 6522. 35444208 10.1038/s41598-022-10522-3 PMC9021310

[acel70358-bib-0047] Suda, M. , I. Shimizu , G. Katsuumi , et al. 2021. “Senolytic Vaccination Improves Normal and Pathological Age‐Related Phenotypes and Increases Lifespan in Progeroid Mice.” Nature Aging 1, no. 12: 1117–1126. 37117524 10.1038/s43587-021-00151-2

[acel70358-bib-0048] Tchkonia, T. , D. E. Morbeck , T. von Zglinicki , et al. 2010. “Fat Tissue, Aging, and Cellular Senescence.” Aging Cell 9, no. 5: 667–684. 20701600 10.1111/j.1474-9726.2010.00608.x PMC2941545

[acel70358-bib-0049] Tchkonia, T. , A. K. Palmer , and J. L. Kirkland . 2021. “New Horizons: Novel Approaches to Enhance Healthspan Through Targeting Cellular Senescence and Related Aging Mechanisms.” Journal of Clinical Endocrinology and Metabolism 106, no. 3: e1481–e1487. 33155651 10.1210/clinem/dgaa728 PMC7947756

[acel70358-bib-0050] Teo, Y. V. , N. Rattanavirotkul , N. Olova , et al. 2019. “Notch Signaling Mediates Secondary Senescence.” Cell Reports 27, no. 4: 997–1007.e5. 31018144 10.1016/j.celrep.2019.03.104 PMC6486482

[acel70358-bib-0051] Tripathi, U. , A. Misra , T. Tchkonia , and J. L. Kirkland . 2021. “Impact of Senescent Cell Subtypes on Tissue Dysfunction and Repair: Importance and Research Questions.” Mechanisms of Ageing and Development 198: 111548. 34352325 10.1016/j.mad.2021.111548 PMC8373827

[acel70358-bib-0052] Tripathi, U. , R. Nchioua , L. G. P. L. Prata , et al. 2021. “SARS‐CoV‐2 Causes Senescence in Human Cells and Exacerbates the Senescence‐Associated Secretory Phenotype Through TLR‐3.” Aging 13, no. 18: 21838–21854. 34531331 10.18632/aging.203560 PMC8507266

[acel70358-bib-0053] Van Der Maaten, L. 2014. “Accelerating t‐SNE Using Tree‐Based Algorithms.” Journal of Machine Learning Research 15: 3221–3245.

[acel70358-bib-0054] Victorelli, S. , H. Salmonowicz , J. Chapman , et al. 2023. “Apoptotic Stress Causes mtDNA Release During Senescence and Drives the SASP.” Nature 622, no. 7983: 627–636. 37821702 10.1038/s41586-023-06621-4 PMC10584674

[acel70358-bib-0055] Wang, T. W. , Y. Johmura , N. Suzuki , et al. 2022. “Blocking PD‐L1‐PD‐1 Improves Senescence Surveillance and Ageing Phenotypes.” Nature 611, no. 7935: 358–364. 36323784 10.1038/s41586-022-05388-4

[acel70358-bib-0056] Wissler Gerdes, E. O. , Y. Zhu , T. Tchkonia , and J. L. Kirkland . 2020. “Discovery, Development, and Future Application of Senolytics: Theories and Predictions.” FEBS Journal 287, no. 12: 2418–2427. 32112672 10.1111/febs.15264 PMC7302972

[acel70358-bib-0057] Wissler Gerdes, E. O. , Y. Zhu , B. M. Weigand , et al. 2020. “Cellular Senescence in Aging and Age‐Related Diseases: Implications for Neurodegenerative Diseases.” International Review of Neurobiology 155: 203–234. 32854855 10.1016/bs.irn.2020.03.019 PMC7656525

[acel70358-bib-0058] Wu, T. , E. Hu , S. Xu , et al. 2021. “clusterProfiler 4.0: A Universal Enrichment Tool for Interpreting Omics Data.” Innovation 2, no. 3: 100141. 34557778 10.1016/j.xinn.2021.100141 PMC8454663

[acel70358-bib-0059] Wyld, L. , I. Bellantuono , T. Tchkonia , et al. 2020. “Senescence and Cancer: A Review of Clinical Implications of Senescence and Senotherapies.” Cancers (Basel) 12, no. 8: 2134. 32752135 10.3390/cancers12082134 PMC7464619

[acel70358-bib-0060] Wyles, S. P. , T. Tchkonia , and J. L. Kirkland . 2022. “Targeting Cellular Senescence for Age‐Related Diseases: Path to Clinical Translation.” Plastic and Reconstructive Surgery 150: 20s–26s. 10.1097/PRS.0000000000009669 PMC9529239 36170432

[acel70358-bib-0061] Xu, M. , E. W. Bradley , M. M. Weivoda , et al. 2017. “Transplanted Senescent Cells Induce an Osteoarthritis‐Like Condition in Mice.” Journals of Gerontology. Series A, Biological Sciences and Medical Sciences 72, no. 6: 780–785. 27516624 10.1093/gerona/glw154 PMC5861939

[acel70358-bib-0062] Xu, M. , T. Pirtskhalava , J. N. Farr , et al. 2018. “Senolytics Improve Physical Function and Increase Lifespan in Old Age.” Nature Medicine 24, no. 8: 1246–1256. 10.1038/s41591-018-0092-9 PMC6082705 29988130

[acel70358-bib-0063] Xu, M. , T. Tchkonia , H. Ding , et al. 2015. “JAK Inhibition Alleviates the Cellular Senescence‐Associated Secretory Phenotype and Frailty in Old Age.” Proceedings of the National Academy of Sciences of the United States of America 112, no. 46: E6301–E6310. 26578790 10.1073/pnas.1515386112 PMC4655580

[acel70358-bib-0064] Xu, Q. , Q. Fu , Z. Li , et al. 2021. “The Flavonoid Procyanidin C1 Has Senotherapeutic Activity and Increases Lifespan in Mice.” Nature Metabolism 3, no. 12: 1706–1726. 10.1038/s42255-021-00491-8 PMC8688144 34873338

[acel70358-bib-0065] Zhang, P. , Y. Kishimoto , I. Grammatikakis , et al. 2019. “Senolytic Therapy Alleviates Aβ‐Associated Oligodendrocyte Progenitor Cell Senescence and Cognitive Deficits in an Alzheimer's Disease Model.” Nature Neuroscience 22, no. 5: 719–728. 30936558 10.1038/s41593-019-0372-9 PMC6605052

[acel70358-bib-0066] Zhang, Y. , G. Parmigiani , and W. E. Johnson . 2020. “ComBat‐Seq: Batch Effect Adjustment for RNA‐Seq Count Data.” NAR Genomics and Bioinformatics 2, no. 3: lqaa078. 33015620 10.1093/nargab/lqaa078 PMC7518324

[acel70358-bib-0067] Zhu, Y. , E. J. Doornebal , T. Pirtskhalava , et al. 2017. “New Agents That Target Senescent Cells: The Flavone, Fisetin, and the BCL‐X(L) Inhibitors, A1331852 and A1155463.” Aging (Albany NY) 9, no. 3: 955–963. 28273655 10.18632/aging.101202 PMC5391241

[acel70358-bib-0068] Zhu, Y. , T. Tchkonia , T. Pirtskhalava , et al. 2015. “The Achilles' Heel of Senescent Cells: From Transcriptome to Senolytic Drugs.” Aging Cell 14, no. 4: 644–658. 25754370 10.1111/acel.12344 PMC4531078

