# Supplementary material for: Senolytic‐Resistant Senescent Cells Have a Distinct SASP Profile and Functional Impact: The Path to Developing Senosensitizers
Source: Aging Cell. 2025 Dec 29;25(1):e70358. doi: 10.1111/acel.70358 (PMC12748526; doi:10.1111/acel.70358)
Supplement: Supplementary file 1 — Appendix S1: acel70358‐sup‐0001‐AppendixS1.zip. [file ACEL-25-e70358-s001.zip › acel70358-sup-0001-AppendixS1/Supplemental Figures1-10.docx]

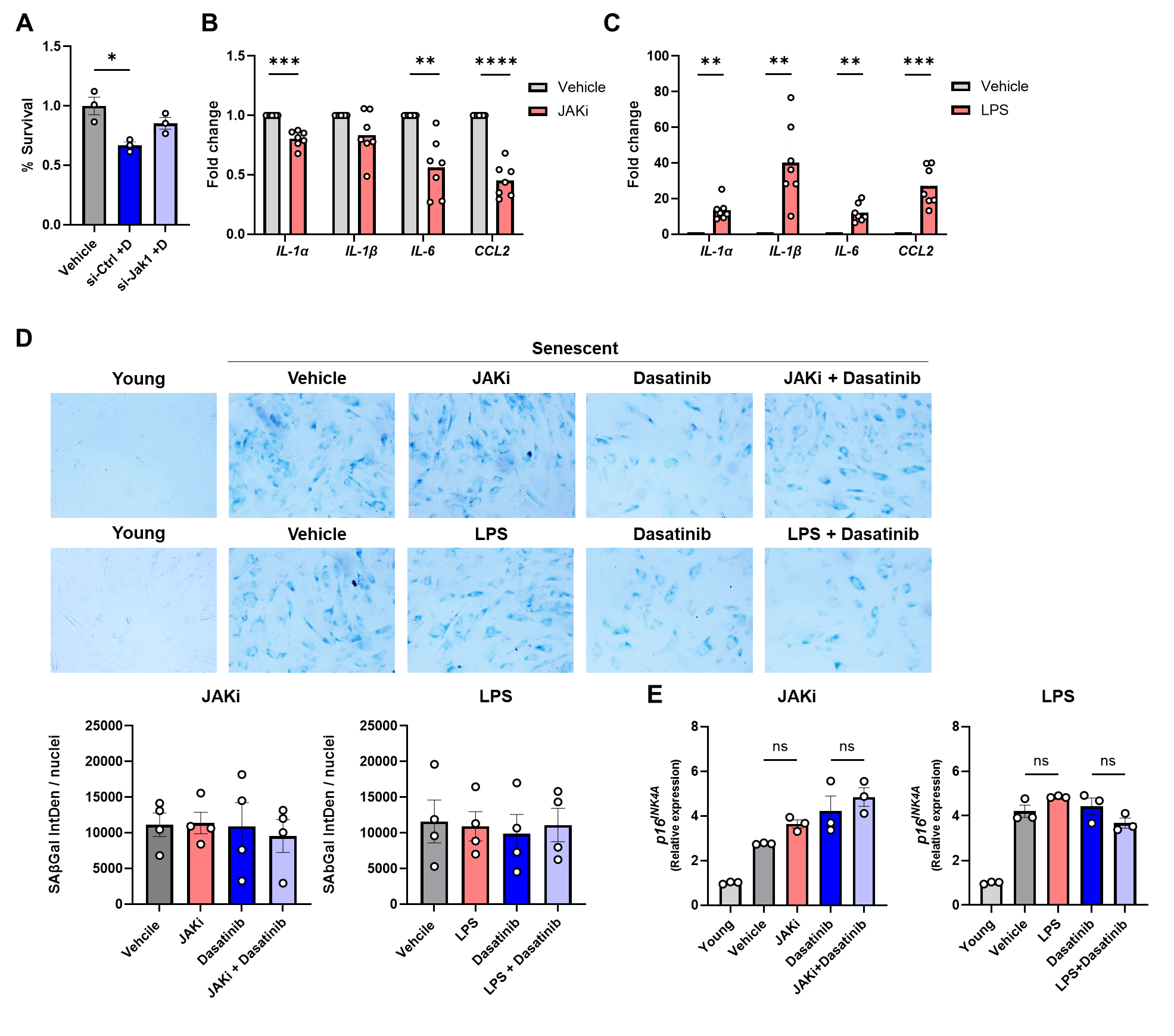


**Fig. S1. Effect of SASP modulation on senescent cell survival and pro-inflammatory factors**. (A) % Survival of senescent preadipocytes. Means +/- SEM, 1-way ANOVA; *post hoc* comparisons by Tukey’s HSD multiple comparison test. (B) JAKi and (C) LPS modulate SASP expression (rt-PCR) of senescent preadipocytes. Data are expressed as a function of vehicle-treated cells. Means +/- SEM, paired, two-tailed Student’s T-tests. (D) Representative images and quantification of SAβgal intensity in senescent preadipocytes. Means +/- SEM, unpaired, 1-way ANOVA with *post hoc* pairwise comparisons by Tukey’s HSD multiple comparison test. (E) *p16^INK4a^ (CDKN2A)* expression in senescent preadipocytes. Data are expressed as a function of non-senescent control cells. Means +/- SEM; unpaired, 1-way ANOVA; *post hoc* comparisons by Tukey’s HSD multiple comparison test.


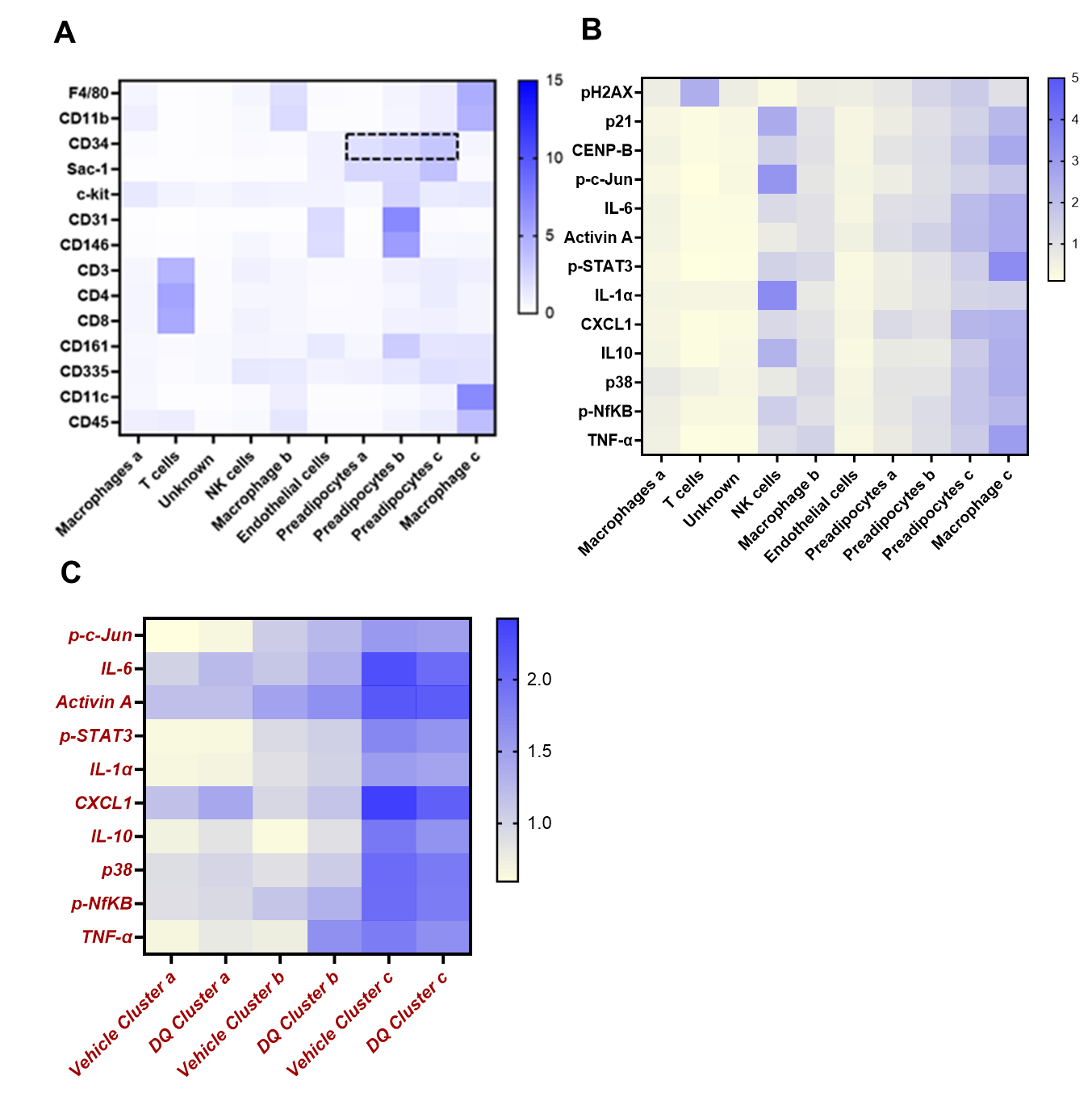


**Fig. S2**. Heatmap of the 10 cell clusters in Fig. 2B with cell identification markers (A) and SASP proteins (B). The preadipocyte cluster is highlighted. (C) SASP profiles in vehicle *vs*. Dasatinib plus Quercetin-treated preadipocytes.

**
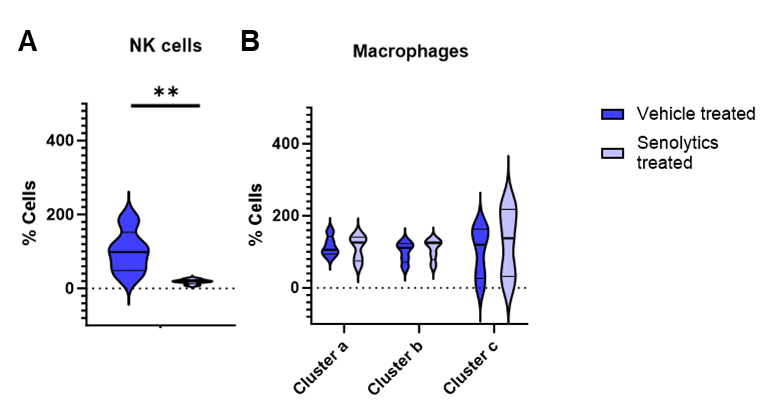
**

**Fig. S3. Senolytic treatment alters NK cell adipose tissue content**. (A) Percent of NK cells in each cluster in vehicle- *vs*. senolytic-treated obese mice. (B) Percent of macrophages in vehicle- *vs*. senolytic-treated obese mice. Means +/- SEM; unpaired two-tailed Mann-Whitney tests.


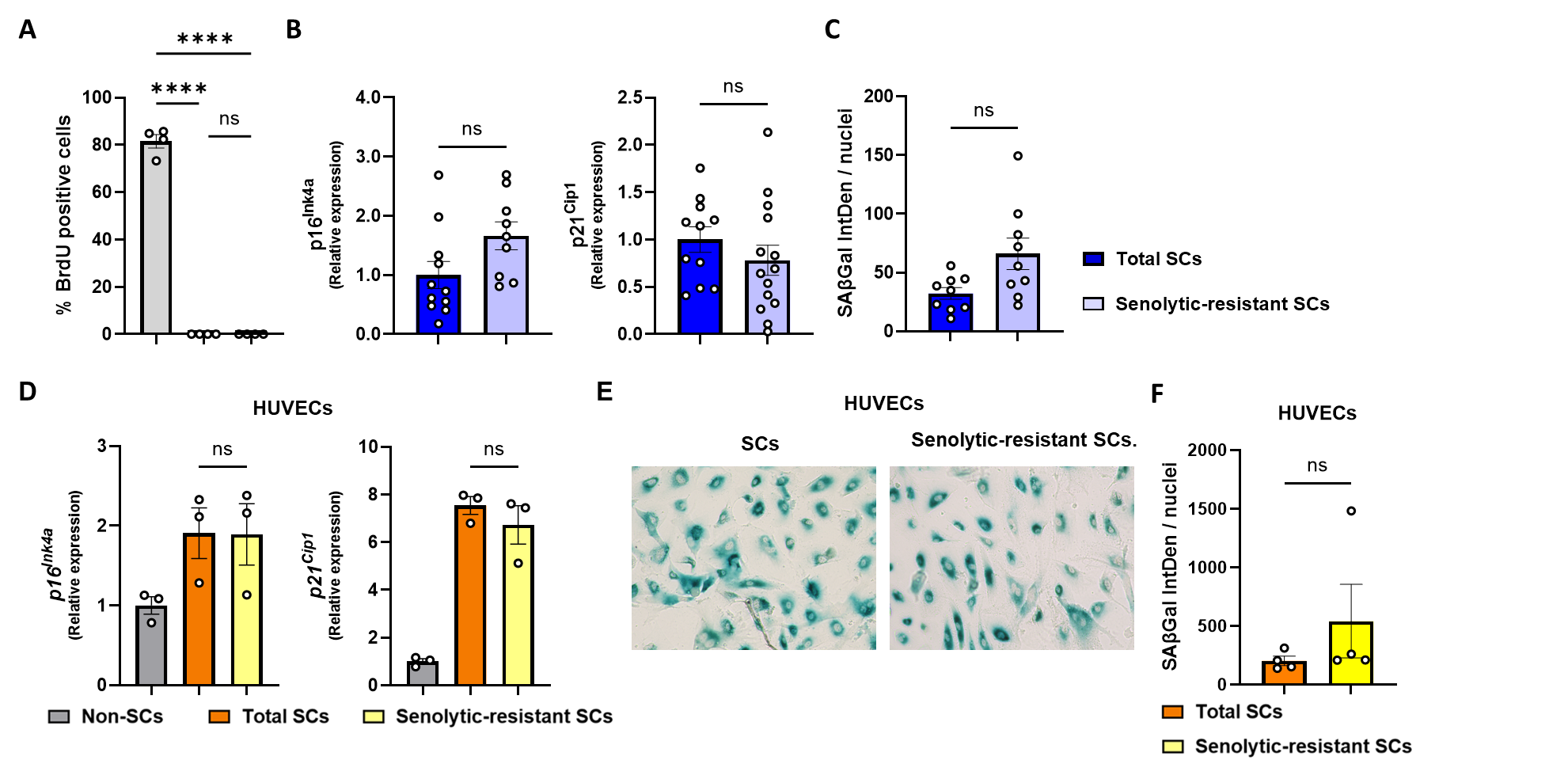


**Fig. S4. Supplemental data related to Fig. 3**. **As in human senescent preadipocytes, senescence markers are similar in the human endothelial cell (HUVEC) senolytic-resistant *vs*. total SC populations**. (A) Quantification of BrdU positive cells in Fig. 3A. (B) Quantification of senescence markers in Fig. 3C. (C) Quantification of SAβgal intensity in Fig. 3E in total *vs*. senolytic (Dasatinib)-resistant senescent preadipocytes. Means +/- SEM; paired or unpaired two-tailed Student’s T-tests. (D) Gene expression for the indicated senescence markers (N=3) in total senescent HUVEC populations *vs*. senolytic-resistant senescent HUVECs. Data are expressed as a function of non-senescent control cells. Means +/- SEM; unpaired, 1-way ANOVA; *post hoc* comparisons by Tukey’s HSD multiple comparison test. (E) Representative images and (F) quantification of SAβgal intensity in senescent HUVECs. Means +/- SEM; paired two-tailed Student’s T-tests.

**Fig. S5. Heat map of SASP factor expression in conditioned media collected from the total senescent cell or senolytic-resistant senescent cell preadipocyte populations (n=3)**. Cells were treated with Dasatinib for 3 days. Multiplex ELISA was performed in conditioned media collected after 72 hrs. after washing out Dasatinib. Relative abundance in senolytic-resistant *vs*. total SCs; results normalized to the mean of vehicle-treated total SCs.


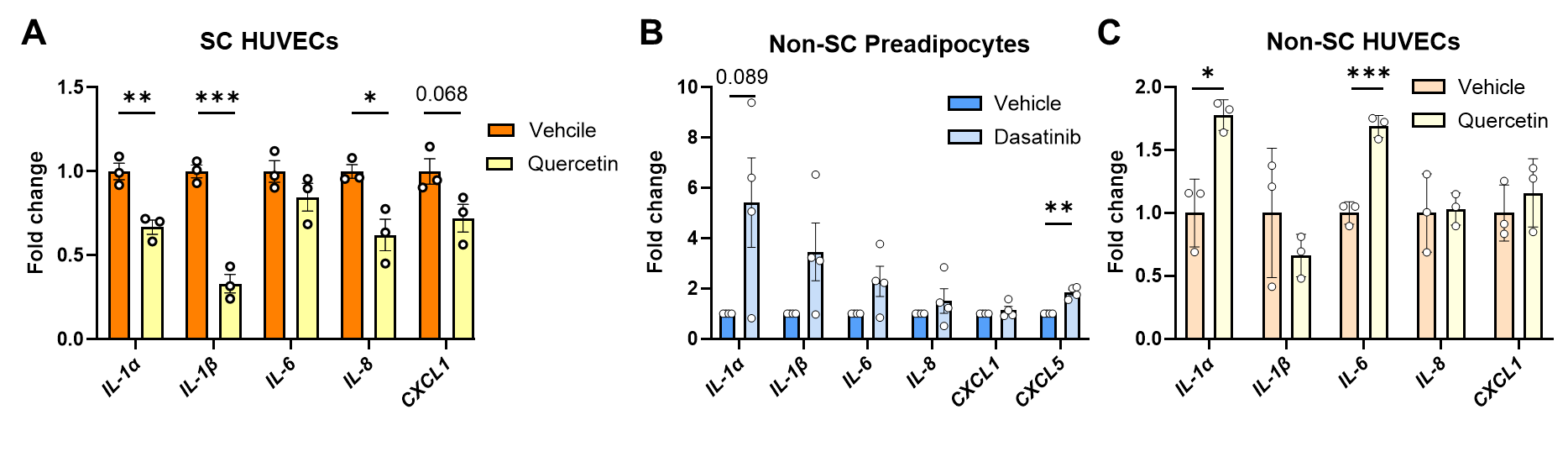


**Fig. S6. Senolytic effects on pro-inflammatory factors in non-senescent or senescent preadipocytes and HUVECs**. (A) SASP factor expression in Quercetin-resistant senescent HUVECs, (B) Dasatinib-resistant non-senescent human preadipocytes, and (C) Quercetin-resistant non-senescent HUVECs expressed as a function of HUVECs treated with vehicle. Data are expressed as a function of vehicle-treated cells; means +/- SEM; unpaired (A, C) or paired (B), two-tailed Student’s T-tests.

**Fig. S7. Cell-free nuclear DNA release by the non-senescent *vs*. the total senescent (senolytic-resistant plus senolytic-sensitive) *vs*. senolytic-resistant human preadipocyte populations**. Abundance of cell-free KRAS DNA in CM prepared from the indicated cell types. Means +/- SEM. No statistically significant differences (ns) were detected among three groups by paired, 1-way ANOVA with *post hoc* pairwise comparisons by Tukey’s HSD multiple comparison test.


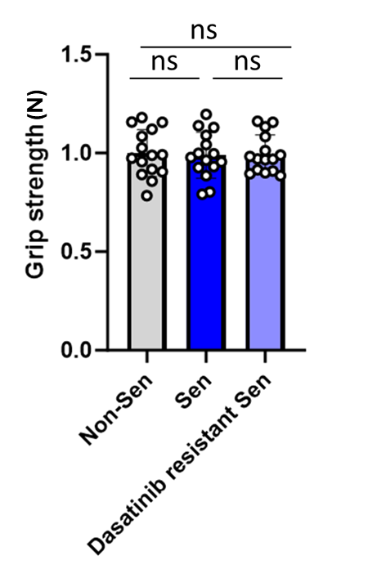


**Fig. S8: Baseline grip strength prior to transplantation of cells**. Base line grip strength in 2-month-old male *SCID-Beige* mice (N=15). Data are shown as means +/- SEM with individual value. No statistically significant differences (ns) among three groups by unpaired two-tailed Student’s T-tests.

**
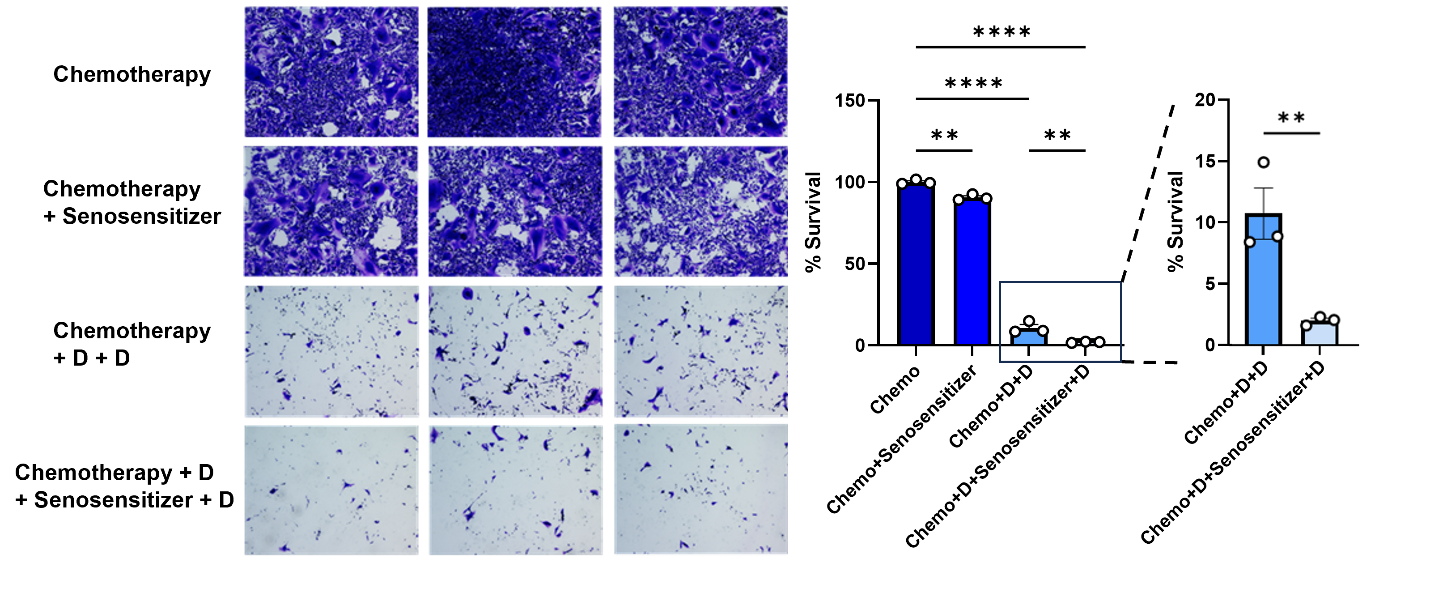
Fig. S9: A “1:2:3:4”-step approach enhances ablation of cultured TNBC cells**. Representative images of surviving (crystal violet+) MDA-MB-231 human breast cancer cells and quantification relative to chemotherapy only-treated cells. Chemo: Cisplatin (1µ/ml), Senosensitizer: TLR3 agonist (polyinosinic-polycytidylic acid) (10 µg/ml), D: Dasatinib (200 nM). This “1:2:3:4” step regimen was repeated 3 times. These findings provide early evidence that a sequential therapeutic approach incorporating chemotherapy, senosensitizers, and senolytics could offer a promising strategy to overcome resistance in aggressive cancers such as TNBC, a possibility that merits further study. Data are shown as means +/- SEM with individual values; 1-way ANOVA; *post hoc* pairwise comparisons by Tukey’s HSD multiple comparison test.


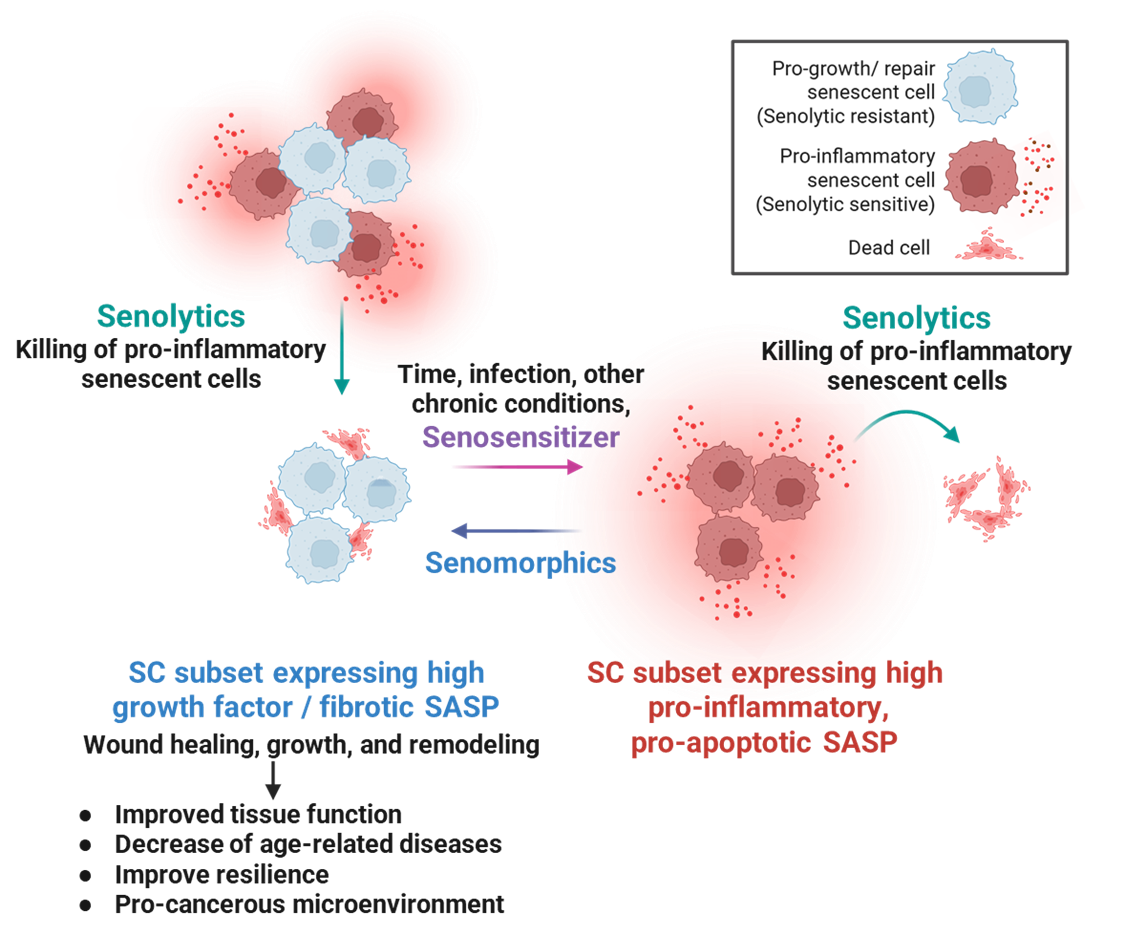


**Fig. S10. Summary and implications**. The SC subtype with a pro-inflammatory, pro-apoptotic, tissue-damaging SASP is susceptible to senolytics. The SC subset not killed by senolytics have a pro-growth/ repair SASP with less mtDNA release and less proclivity to cause physical dysfunction following transplantation into younger individuals than the total SC population. On the one hand, agents such as Ruxolitinib can attenuate the pro-inflammatory SASP, making SCs become resistant to senolytics. On the other, insults such as infections (exposure to PAMPs) can accentuate inflammatory SASP features within hours [56], at least *in vitro*. Therefore, we propose it may be feasible to develop agents, “senosensitizers”, that induce senolytic-resistant SCs to be converted into SCs that are susceptible to senolytics. Sequencing senosensitizers with senolytics could enable removal of a pool of relatively silent SCs harbored in apparently asymptomatic individuals, which could otherwise be activated within hours by infections or other insults into deleterious cells.
